# Unveiling Immune-related feature genes for Alzheimer’s disease based on machine learning

**DOI:** 10.3389/fimmu.2024.1333666

**Published:** 2024-06-10

**Authors:** Guimei Zhang, Shuo Sun, Yingying Wang, Yang Zhao, Li Sun

**Affiliations:** ^1^ Department of Neurology and Neuroscience Center, The First Hospital of Jilin University, Jilin University, Changchun, China; ^2^ Cognitive Center, Department of Neurology, The First Hospital of Jilin University, Jilin University, Changchun, China; ^3^ Department of Urology, The Affiliated Hospital of Changchun University of Chinese Medicine, Changchun University of Chinese Medicine, Changchun, China; ^4^ The Second Department of Pediatrics, The First Hospital of Jilin University, Jilin University, Changchun, China

**Keywords:** Alzheimer’s disease, feature genes, immune regulation, machine learning, small molecule compounds, therapeutic targets

## Abstract

The identification of diagnostic and therapeutic biomarkers for Alzheimer’s Disease (AD) remains a crucial area of research. In this study, utilizing the Weighted Gene Co-expression Network Analysis (WGCNA) algorithm, we identified RHBDF2 and TNFRSF10B as feature genes associated with AD pathogenesis. Analyzing data from the GSE33000 dataset, we revealed significant upregulation of RHBDF2 and TNFRSF10B in AD patients, with correlations to age and gender. Interestingly, their expression profile in AD differs notably from that of other neurodegenerative conditions. Functional analysis unveiled their involvement in immune response and various signaling pathways implicated in AD pathogenesis. Furthermore, our study demonstrated the potential of RHBDF2 and TNFRSF10B as diagnostic biomarkers, exhibiting high discrimination power in distinguishing AD from control samples. External validation across multiple datasets confirmed the robustness of the diagnostic model. Moreover, utilizing molecular docking analysis, we identified dinaciclib and tanespimycin as promising small molecule drugs targeting RHBDF2 and TNFRSF10B for potential AD treatment. Our findings highlight the diagnostic and therapeutic potential of RHBDF2 and TNFRSF10B in AD management, shedding light on novel strategies for precision medicine in AD.

## 1 Introduction

Alzheimer’s disease (AD) is the most common form of age-related dementia and is defined as a progressive neurodegenerative disorder, which predominantly manifests as an impairment of cognitive function, particularly anterograde episodic memory, accompanied by declines in visuospatial, language, and executive functions (1). Data from the World Alzheimer’s Disease Report 2023 suggests that the number of people with dementia worldwide will increase from 55 million in 2019 to 139 million in 2050 (2). Moreover, with rapidly aging populations throughout, the number of dementia patients will further increase. As the leading cause of dementia (accounting for 60–80% of all cases), AD prevalence is also on the rise, posing an ever greater socioeconomic and healthcare burden.

Genetic predisposition has been established as a major risk factor for AD. Mutations in several genes, including amyloid precursor protein, presenilin 1, presenilin 2, and the ϵ4 allele of apolipoprotein E, have been implicated in AD pathogenesis (3). Further, a number of immune-related genes have been identified as strongly associated with AD (4). Genome-wide association studies (GWAS) have also gained ground with increasing sample sizes and inexpensive gene chip technology, which has expanded our understanding of the genetic architecture of AD (5). Integration of AD GWAS with tissue- and cell-type-specific epigenetic annotations has suggested a modulatory role for various immune components in AD susceptibility. In addition to microglia, which have been at the center of AD research, the adaptive immune cells T and B lymphocytes have been shown to play a key role in regulating AD pathology (6–8), which influences AD progression and disease severity. Therefore, understanding the intricate relationship between immunity and AD would be a promising strategic direction that could contribute to a deeper insight into the pathogenesis of AD and facilitate the discovery of novel immune-related biodiagnostic markers. Targeted modulation of the activities of different immune components will also facilitate the development of new therapeutic interventions.

The involvement of different cellular and molecular subtypes, pathways and networks in the pathogenesis of AD has limited the understanding of the heterogeneity and complexity of AD and has significantly hindered progress in the diagnosis and treatment of AD. In recent years, with the increasing development of multi-omics such as genetics and genome projects, data mining research has shown its importance in AD research. Bioinformatics analysis is a discipline based on the combination of computer science and bioinformatics tools, methods and techniques (9), where collected DNA, mRNA and protein data can be analyzed and organized as required. It can reveal potential modes of action and disease mechanisms at the molecular level, depending on the research objectives. However, effective integration of multi-omics data is particularly important due to small sample sizes and redundant data. Machine learning (ML) combined with bioinformatics data can overcome the limitations of data set size and build predictive models for disease occurrence and progression by effectively mining large amounts of data (10, 11). Based on the manipulation of multi-omics data, ML uses different algorithms to extract unique insights from the data to target different immune cells and immune-related genes, and successfully achieves the precise analysis of the key functions of immune-related signature genes in the pathogenesis of AD.

This study delves into the expression of novel immune-related genes in AD, shedding light on their pivotal roles in immune processes and pathways. By analyzing immune cell infiltration and clinical correlations, the research uncovers valuable insights into AD pathogenesis. Furthermore, the development of a diagnostic nomogram based on these genes offers a promising tool for AD diagnosis. The study also explores potential treatments by identifying small molecule compounds through advanced technology. These findings not only deepen our understanding of AD but also provide new avenues for targeted interventions and future clinical trials in the quest for effective AD therapies.

## 2 Materials and methods

### 2.1 Data acquisition and processing

The GEO database (12) is a public repository for expression data, including microarrays, second-generation sequencing, and high-throughput sequencing. We selected “Alzheimer’s disease”, “Homo sapiens”, and “expression profiling by array” as MeSH search terms. Datasets with significant age differences between groups were excluded. The series matrix files for GSE33000 (310 AD brain samples and 157 normal brain samples) (13), GSE118553 (167 AD brain samples and 100 normal brain samples) (14), GSE44772 (387 AD brain samples and 303 normal brain samples) (15), and GSE122063 (24 AD brain samples and 22 normal brain samples) (16) were selected and downloaded, with the probe names converted to gene symbols using R software (17, 18). We then use the “ComBat” function to batch correct the merged dataset (GSE44772, GSE118553, GSE122063) (19). In cases where datasets had missing values, we employed multiple imputation to handle these missing values. This involved the use of weighted average from k-nearest neighbors approach. The sample collection for these datasets is shown in **Supplementary Table S1**. To identify genes that are specifically expressed in AD patients, we downloaded series matrix files from the GEO database for Parkinson’s disease (GSE20168, GSE7621, GSE20291, and GSE20292), frontotemporal dementia (GSE195872), dementia with Lewy bodies (GSE150696), and Huntington’s disease (GSE33000). For our analysis, we standardized the data using the “normalizeBetweenArrays” function and “Log2”.

### 2.2 Identification of differentially expressed genes (DEGs)

The DEGs analysis between AD patients and controls were screened using the lmFit and eBayes functions in the limma R package. DEGs were considered significant if the |log fold change (FC)| was greater than log1.2 and the adjusted p-value was less than 0.01. To visualize the results, volcano plots and heatmap plots were created using the R packages “ggplot” and “pheatmap”.

### 2.3 Feature genes generated from machine learning-based WGCNA algorithm

Genes were screened for WGCNA analysis using thresholds of |logFC| > 0.05 and adjusted p-value < 0.01 (20). To ensure a reliable scale-free network, we set a threshold of scale-free fit R2 > 0.85. The “blockwiseModules” function was utilized with the following parameters: minModuleSize = 80, mergeCutHeight = 0.2, and TOMType = “unsigned” for network construction and module detection. The identification of the key module was based on the criteria of the greatest gene significance (GS) and the highest correlation coefficient between the module and the trait. In our study, feature genes were defined as genes within a module exhibiting high connectivity and regarded as functionally significant (21). Specifically, for this analysis, feature genes were characterized by having a GS and module membership (MM) within the key module.

### 2.4 Functional enrichment and immune cell infiltration analysis for AD patients and feature genes

To investigate the biological functions related to AD patients, we conducted gene set variation analysis (GSVA). The gene sets used in this study were the hallmark gene set (h.all.v2023.1.Hs.symbols) and Kyoto Encyclopedia for Genes and Genomes (KEGG) gene set (c2.cp.kegg.v2023.1.Hs.symbols) from the Molecular Signatures Database (MSigDB) V7.0 database. These gene sets were converted to scores (z-scores) using the single-sample gene set enrichment analysis (ssGSEA) method of the GSVA function (22, 23). Additionally, given the strongest GS and highest correlation coefficients between key modules and AD patients, we focused on the genes within the key modules for Gene Ontology (GO) and KEGG analyses. The GO method serves as a fundamental bioinformatics tool for gene annotation, classifying genes into categories such as biological process (BP), molecular function (MF), and cellular component (CC). For these analyses, we utilized the “clusterProfiler” R package (24, 25). To gain knowledge about immune cell infiltration in AD patients, we used the CIBERSORT algorithm (26) to assess the proportions of 22 subtypes of infiltrating immune cells. Simultaneously, the signatures of 64 immune and stromal cells were converted into enrichment scores using the “xCell” R package (27).

We categorized AD patients into high and low expression groups based on the median expression levels of feature genes (28). To explore the biological functions of these feature genes, we performed GSVA analysis. We also used the CIBERSORT and xCell algorithms to evaluate the immune cell infiltration patterns within the high and low expression groups of these genes. In addition, we employed the GeneMANIA database (29, 30) (http://www.genemania.org/) to identify genes functionally related to the feature genes and to predict their potential functions. Statistical significance was set at p < 0.05.

### 2.5 Multivariable classifier performance assessment and validation

To evaluate the potential of feature genes in differentiating between AD patients and controls, we calculated the highest area under the receiver operating curve (AUROC) using the “pROC” package (31). In order to enhance the diagnostic ability of identifying AD patients, we constructed a multivariable classifier by combining feature genes with clinical features. We randomly divided the GSE33000 dataset into a training dataset and an internal validation dataset in an 8:2 ratio. Furthermore, we performed external validation to assess the accuracy of the diagnostic model. To validate the diagnostic performance of the multivariate model, we performed diagnostic prediction in GSE44772, GSE118553, GSE122063, and meta-cohort (overall dataset).

### 2.6 Nomogram, calibration curve analysis, decision curve analysis, and clinical impact curve of multivariate diagnostic classifier

A nomogram, a graphical tool for efficient approximation of complex calculations, was constructed in this study using the “rms” R package (32). The nomogram model incorporated selected signatures from a diagnostic multivariable classifier and aimed to predict the occurrence of AD patients. To assess the nomogram’s performance, the concordance index (C-index) was calculated using a bootstrap method with 1000 resamples, providing a measure of discrimination. Furthermore, CCA was plotted to compare the prediction probabilities of the nomogram with observed rates. The clinical utility of the model was assessed using DCA curves. DCA takes into account the relative value of benefits and harms associated with the prediction model, thereby surpassing the limitations posed by traditional statistical metrics (33). The CIC is a visual tool utilized to assess and present the performance of diagnostic models or diagnostic genes across various threshold probability ranges. The predictive value of the diagnostic model was evaluated using the “rmda” R package in this study.

### 2.7 Drug screening and docking

We conducted a screening process to identify potential small molecule drugs. Firstly, we searched for co-expressed genes that showed a correlation greater than 0.85 with feature genes in the gene expression matrices of AD patients. Next, we used the CMap database (https://clue.io/) to identify drugs that displayed significant correlations with these genes (34). We employed connectivity scores (CS) and FDR to measure correlation and statistical significance, respectively. The CS close to -1, on a scale ranging from +1 to -1, indicated potential therapeutic value. For our selection criteria, we set a FDR threshold of < 0.05 and a CS threshold of < -0.8. To investigate the interaction between small molecule compounds and feature genes, we employed molecular docking. First, the 2D structures of small molecule ligands were obtained from the PubChem database (http://pubchem.ncbi.nlm.nih.gov/) and converted to 3D structures using Chem Office 20.0 software, and saved as mol2 files. Next, protein targets with high-resolution crystal structures were selected from the RCSB PDB database (http://www.rcsb.org/) for molecular docking, and the proteins were prepared by removing water and phosphate groups using PyMOL 2.6.0 software, and saved as PDB files. The compounds were subjected to energy minimization using the Molecular Operating Environment 2019 software, and the target proteins were preprocessed to identify active pockets. Finally, molecular docking was performed using MOE 2019. The binding activity of the compounds was assessed based on the binding energy (BE), and the results were visually analyzed using PyMOL 2.6.0 and Discovery Studio 2019 software. Typically, docking energies less than -4.25 kcal/mol indicate some degree of binding activity, less than -5.0 kcal/mol indicate good binding activity, and less than -7.0 kcal/mol indicate strong binding activity.

### 2.8 Statistical analysis

All statistical analyses were performed using version 4.2.3 of the R software. The lmFit and eBayes functions from the “limma” R package were utilized for comparing gene differential expression. Non-parametric tests were used for comparison of immune cell distributions and functional gene set conversion z-scores between groups. Spearman or pearson correlation was used to assess the correlation. A probability level of 0.05 was considered statistically significant for all analyses.

## 3 Results

### 3.1 Feature genes selected using WGCNA algorithm

**Figure 1:**
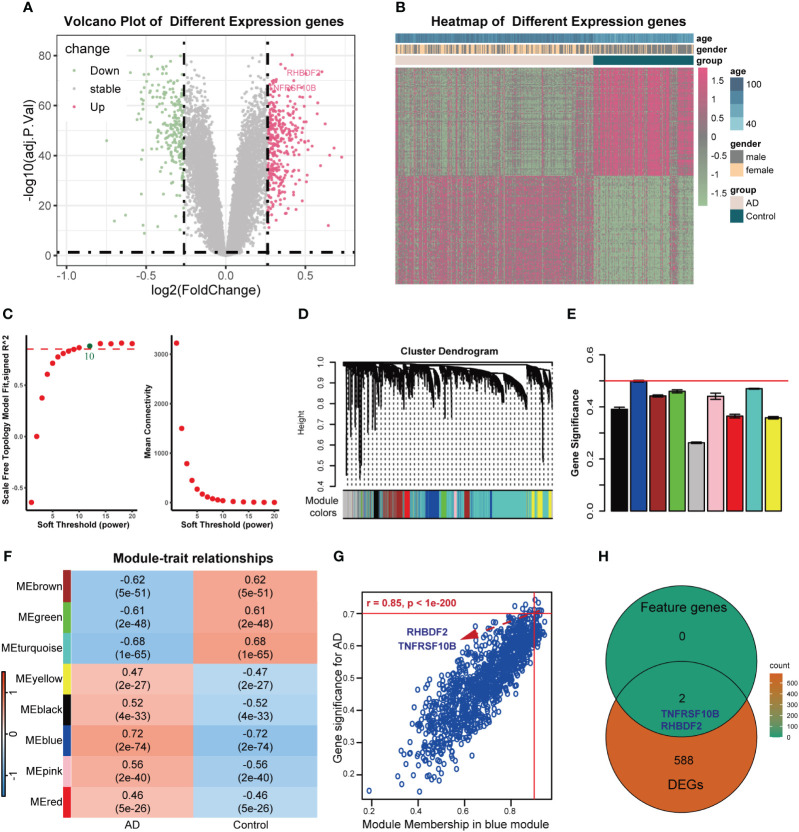
Feature genes selected using WGCNA algorithm in GSE33000 dataset. **(A)** Volcano plot for DEGs between AD patients and controls. **(B)** Heatmap of the 100 significantly upregulated and downregulated genes. **(C)** Analysis of the scale-free topology model fit index for soft threshold powers (left) and the mean connectivity for soft threshold powers (right). **(D)** The cluster dendrogram of genes in GSE33000 dataset. **(E)** The bar chart of gene significance for each module. **(F)** Heatmap of modules correlating with clinical traits. **(G)** Identification of feature genes (GS>0.70, MM>0.92). **(H)** Venn diagrams of feature genes and DEGs.

The overall design of this study was shown in **Supplementary Figure S1**. After comparing AD samples with control samples in the GSE33000 dataset, we successfully identified 590 DEGs. Among these DEGs, 342 were upregulated, and 248 were downregulated. The DEGs were visualized in a volcano plot (**Figure 1A**), and the top 100 significantly upregulated and downregulated DEGs were displayed in a heatmap (**Figure 1B**). Constructing the weighted correlation network with the genes screened from the GSE33000 dataset, we selected a soft threshold power of 10 when a scale-free fit R2 of 0.85 was applied (**Figure 1C**). Subsequently, we constructed 9 co-expression modules (**Figures 1D, E**), with the largest module (turquoise) containing 4,424 genes and the smallest module (pink) containing 136 genes. To identify key modules, we calculated the correlation between modules and AD (**Figure 1F**). The blue module displayed the highest GS and exhibited a strong correlation with AD (r = 0.72, p-value = 2e-74). MM in the blue module (r = 0.85, p < 1e-200) exhibited a significant correlation with GS for AD (**Figure 1G**). In the blue module, a total of 1191 genes were found. By applying cut-off criteria of GS > 0.70 and MM > 0.92, we identified RHBDF2 and TNFRSF10B as feature genes. These two genes are also DEGs, as shown in **Figure 1H**.

### 3.2 Functional analysis of feature genes

**Figure 2:**
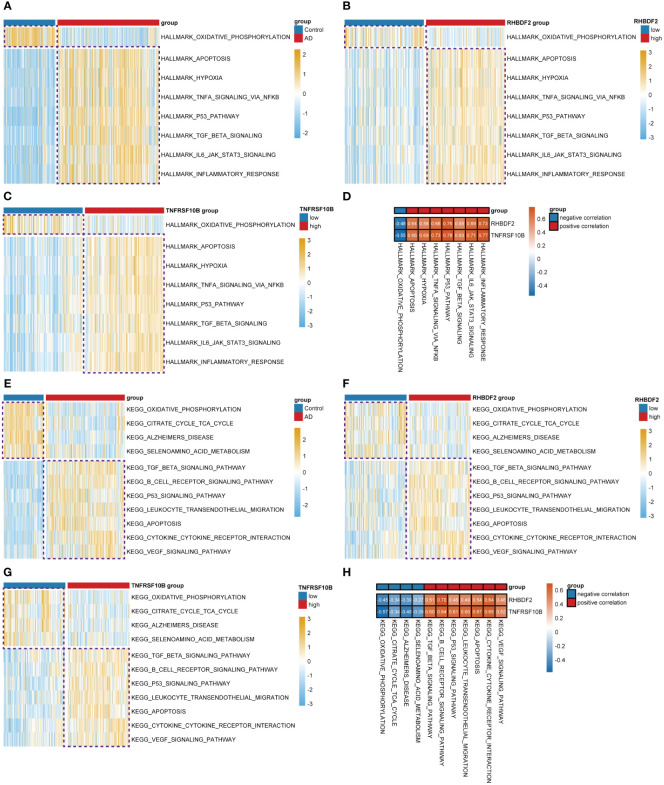
Differential analysis of the z-values for the conversion of hallmark and KEGG gene sets based on GSVA. **(A)** Heatmap for differential analysis of z-scores (hallmark) in AD patients and controls. **(B, C)** Heatmap for differential analysis of z-scores (hallmark) with high and low expression of RHBDF2, TNFRSF10B in AD patients. **(D)** Heatmap of correlation between feature genes and z-scores (hallmark). **(E)** Heatmap for differential analysis of z-scores (KEGG) in AD patients and controls. **(F, G)** Heatmap for differential analysis of z-scores (KEGG) with high and low expression of RHBDF2, TNFRSF10B in AD patients. **(H)** Heatmap of correlation between feature genes and z-scores (KEGG).

To delve further into the potential biological mechanisms of AD, we conducted GSVA using hallmark and KEGG gene sets from MSigDB. We observed that in the high expression groups of RHBDF2 (**Figure 2B**) and TNFRSF10B (**Figure 2C**), oxidative phosphorylation was inhibited, while hallmark biological functions such as apoptosis, hypoxia, TNFα signaling via NF-κB, P53 pathway, TGF-β signaling, IL6-JAK-STAT3 signaling, and inflammatory response were upregulated, aligning with changes seen in AD (**Figure 2A**). Similarly, in our study utilizing KEGG gene sets, we found that oxidative phosphorylation, citrate cycle (TCA cycle), Alzheimer’s disease, and selenoamino acid metabolism were inhibited in the high expression groups of RHBDF2 (**Figure 2F**) and TNFRSF10B (**Figure 2G**). Meanwhile, TGF-β signaling, B cell receptor signaling pathway, P53 signaling pathway, leukocyte transendothelial migration, apoptosis, cytokine-cytokine receptor interaction, and VEGF signaling pathway were upregulated, consistent with changes in AD (**Figure 2E**). GeneMANIA analysis revealed that RHBDF2 is involved in epidermal growth factor receptor and ERBB signaling pathways, interacting with RHBDF1, ADAM17, and EGF (**Supplementary Figure S2**, **Supplementary Table S2**), suggesting RHBDF2’s potential involvement in multiple immune response pathways related to AD. TNFRSF10B, on the other hand, is mainly involved in extrinsic apoptosis signaling pathways, interacting with TNFSF10, FAS, FADD, and CASP8 to form the death-inducing signaling complex (**Supplementary Figure S2**, **Supplementary Table S2**), potentially exacerbating immune cascade reactions in AD through inducing cell apoptosis. Our findings indicate a strong correlation between the expression of RHBDF2 and TNFRSF10B with immune-related biological functions (**Figures 2D, H**), suggesting their involvement in immune processes closely linked to AD. Furthermore, functional enrichment analysis of the blue module significantly associated with AD in WGCNA corroborated our hypothesis (**Supplementary Figure S3**), verifying the implications of our observations.

### 3.3 Association of feature genes expression with immune infiltration

**Figure 3:**
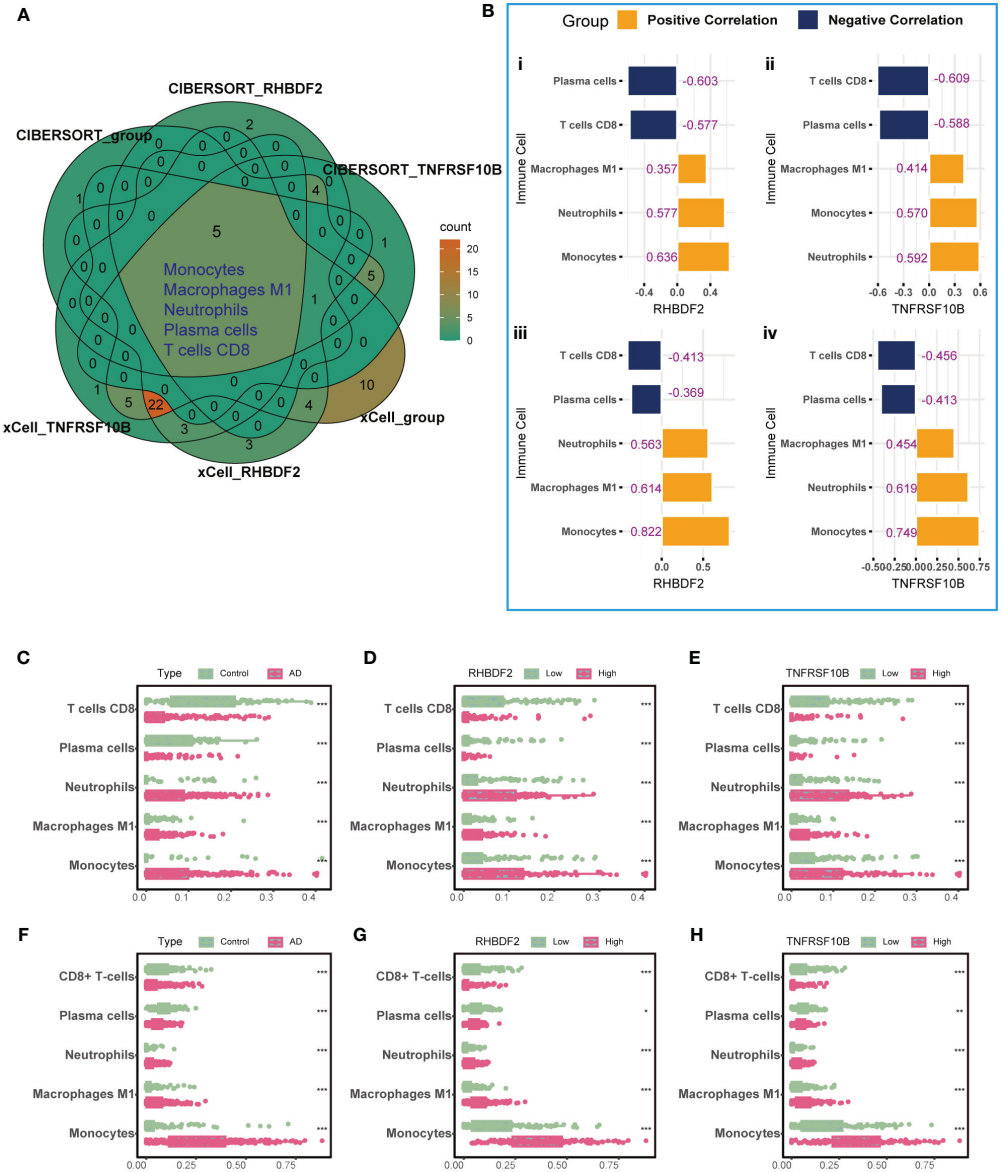
Immune cell infiltration analysis using CIBERSORT and xCell algorithms. **(A)** Five significantly altered immune cell types were identified using the CIBERSORT and xCell algorithms comparing AD patients to controls and to groups with high and low RHBDF2 and TNFRSF10B expression. **(B)** Correlation analysis of RHBDF2 and TNFRSF10B expression with five immune cell types. Correlation analysis based on CIBERSORT algorithm (i, ii) and correlation analysis based on xCell algorithm (iii, iv). The proportions of the five immune cell types were compared between AD patients and controls **(C, F)** and between groups with high and low expression of RHBDF2 **(D, G)** and TNFRSF10B **(E, H)** using the CIBERSORT **(C-E)** and xCell **(F-H)** algorithms. The purple numbers in the **(B)** indicate the correlation coefficients. *p < 0.05, **p < 0.01, and ***p < 0.001.

To elucidate the link between RHBDF2 and TNFRSF10B with infiltrating immune cells in AD, we utilized CIBERSORT (**Supplementary Figure S4**) and xCell (**Supplementary Figure S5**) algorithms to assess immune cell profiles across various cohorts: AD patients versus controls, high versus low RHBDF2 expression, and high versus low TNFRSF10B expression. Our analysis revealed significant changes in five immune cell types - monocytes, neutrophils, M1 macrophages, plasma cells, and CD8+ T cells (**Figure 3A**). Specifically, CD8+ T cells and plasma cells exhibited decreased proportions in the AD, high RHBDF2 expression, and high TNFRSF10B expression groups as per CIBERSORT analysis, while neutrophils, M1 macrophages, and monocytes showed increased proportions in these groups (**Figures 3C-E**). These trends were consistently observed using the xCell algorithm (**Figures 3F-H**). Additionally, correlation analysis illustrated a negative correlation between CD8+ T cells, plasma cells, and RHBDF2/TNFRSF10B expression, and a positive correlation between neutrophils, M1 macrophages, monocytes, and RHBDF2/TNFRSF10B expression (**Figure 3B**). These findings underscore the significant association of RHBDF2 and TNFRSF10B with innate and adaptive immune cells, offering valuable insights into their immunomodulatory roles.

### 3.4 Expression of RHBDF2 and TNFRSF10B association with age and gender

**Figure 4:**
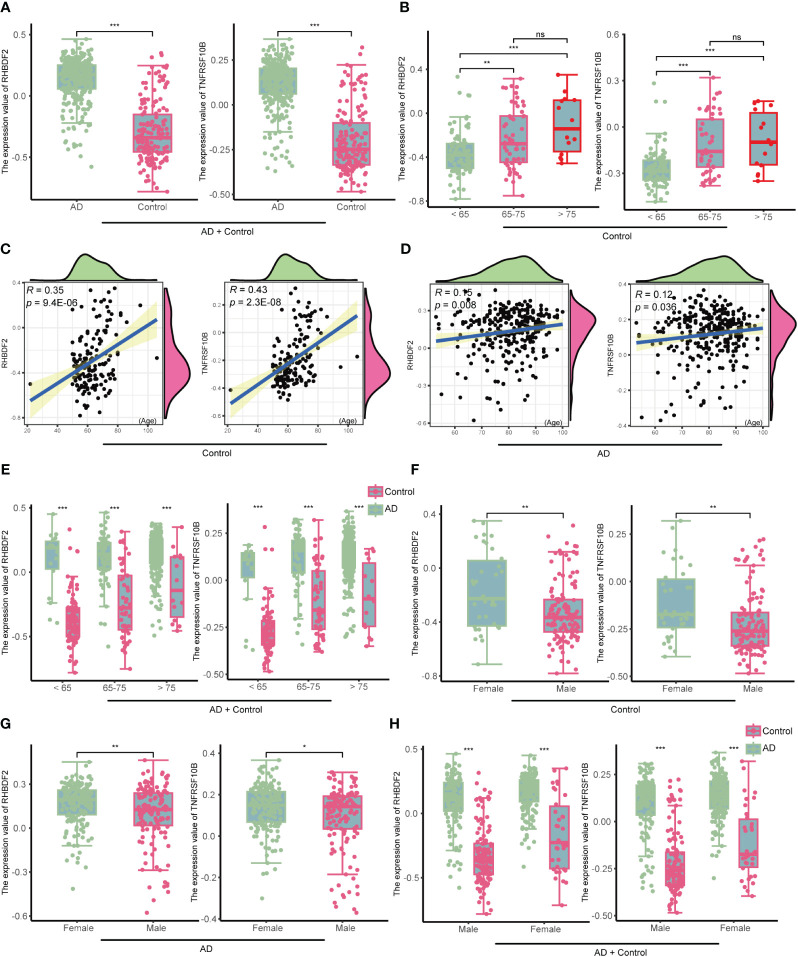
The expression of RHBDF2 and TNFRSF10B in the GSE33000 dataset was associated with age and gender. **(A)** Differential analysis of RHBDF2 and TNFRSF10B expression between the AD group and the control group. **(B)** Differential analysis of RHBDF2 and TNFRSF10B expression across different age groups in the control group. Correlation analysis of RHBDF2 and TNFRSF10B expression with age in the control group **(C)** and the AD group **(D)**. **(E)** Differential analysis of RHBDF2 and TNFRSF10B expression between the AD group and the control group across different age groups. Differential analysis of RHBDF2 and TNFRSF10B expression between genders in the control group **(F)** and the AD group **(G)**. **(H)** Differential analysis of RHBDF2 and TNFRSF10B expression between AD group and control group by gender. *p < 0.05, **p < 0.01, ***p < 0.001, and ns indicates no statistical significance.

In the GSE33000 dataset, a comparative analysis disclosed a significant upregulation of RHBDF2 and TNFRSF10B expression levels in AD patients, as opposed to controls (all p < 0.001, **Figure 4A**). This upregulation trend was further affirmed in the merged dataset comprising GSE44772, GSE118553, and GSE122063 (**Supplementary Figure S6A**). To delve deeper into the potential influence of age and gender on the expression of RHBDF2 and TNFRSF10B in healthy brains, a meticulous stratified analysis was executed on the control samples. The control group was categorized into three distinct age subgroups: <65 years, 65–75 years, and >75 years. This analysis unveiled statistically significant distinctions in the expression levels of RHBDF2 and TNFRSF10B among these subgroups. Noteworthy, the expression levels in the subgroup aged <65 years were significantly lower compared to the other two subgroups. While the expression levels in the 65–75 years subgroup were lower than those in the >75 years subgroup, the variance was not statistically significant (**Figure 4B**).

These age-specific trends in RHBDF2 and TNFRSF10B expression levels in GSE33000 were further validated in the merged dataset (**Supplementary Figure S6B**). Additional correlation analysis indicated a positive correlation between the expression levels of RHBDF2 and TNFRSF10B with age (r = 0.35, p = 9.4e-06; r = 0.43, p = 2.3e-08; **Figure 4C**). Similarly, a positive correlation between the expression levels of both genes and age was observed in the control samples of the merged dataset (r = 0.20, p = 2.4e-05; r = 0.19, p = 4e-05; **Supplementary Figure S6C**). These outcomes propose an increment in the expression of both genes with age. Furthermore, an examination of the expression of these two genes concerning age in the AD group also exhibited a similar increase with age (r = 0.15, p = 0.008; r = 0.12, p = 0.036; **Figure 4D**). Notably, RHBDF2 and TNFRSF10B expression levels were higher in the AD group compared to the control group, regardless of age (**Figure 4E**). These findings were replicated in the merged dataset (**Supplementary Figures S6D, E**), implying a plausible involvement of these genes in the pathophysiology of AD.

Gender analysis unveiled significantly elevated expression levels of RHBDF2 and TNFRSF10B in the female control group compared to the male control group (p = 0.0026, p = 0.0015, respectively, **Figure 4F**). A similar pattern was noted in the AD group, with female patients showing higher expression levels than males (p <0.01, p < 0.05, respectively, **Figure 4G**). However, regardless of gender, the AD group consistently manifested significantly higher expression levels compared to the control group (all p <0.001, **Figure 4H**). These results were further confirmed in the merged dataset (**Supplementary Figures S6F-H**), underscoring a plausible association of RHBDF2 and TNFRSF10B in AD, with notable implications for age and gender.

### 3.5 Expression of RHBDF2 and TNFRSF10B in different brain regions

**Figure 5:**
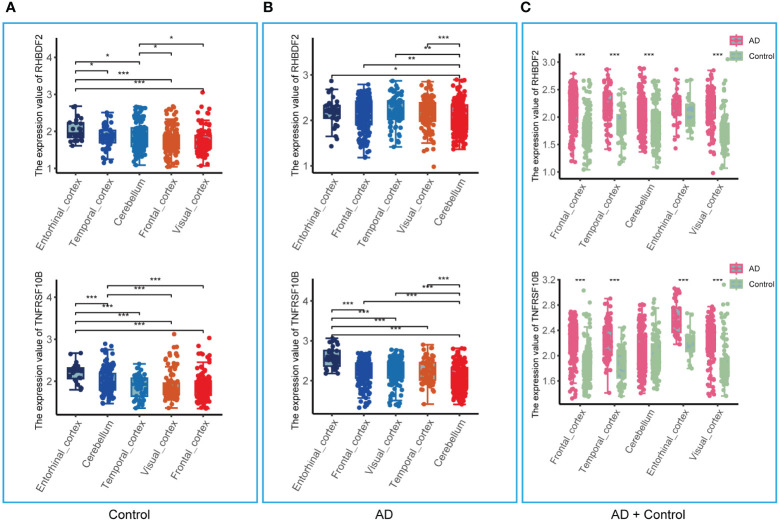
Expression of RHBDF2 and TNFRSF10B in different brain regions in the merged dataset. **(A)** Expression of RHBDF2 (top) and TNFRSF10B (bottom) in different brain regions in the control group. **(B)** Expression of RHBDF2 (top) and TNFRSF10B (bottom) in different brain regions in the AD group. **(C)** Differential analysis of RHBDF2 (top) and TNFRSF10B (bottom) expression in different brain regions between AD and control groups. *p < 0.05, **p < 0.01, and ***p < 0.001.

The analysis in the GSE33000 dataset, limited to the frontal cortex data, prompted an evaluation of the brain region specificity of RHBDF2 and TNFRSF10B expression. This assessment was conducted using a merged dataset encompassing data from five brain regions: entorhinal cortex, cerebellum, temporal cortex, visual cortex, and frontal cortex. Upon scrutiny of the control group data, it was observed that the expression levels of RHBDF2 and TNFRSF10B varied significantly across brain regions, with the most pronounced expression observed in the entorhinal cortex (**Figure 5A**). This brain region specificity was similarly evident in the AD group, with the entorhinal cortex displaying the highest expression of these genes (**Figure 5B**).

Further comparison of RHBDF2 and TNFRSF10B expression levels between AD patients and controls (**Figure 5C**) revealed elevated expression levels in the AD group across multiple brain regions. Specifically, in the frontal cortex, temporal cortex, cerebellum, and visual cortex, RHBDF2 exhibited significantly higher expression in the AD group compared to the control group. While the difference in the entorhinal cortex did not reach statistical significance, there was a discernible increasing trend in expression in the AD group. Moreover, TNFRSF10B expression in the frontal cortex, temporal cortex, entorhinal cortex, and visual cortex of the AD group was significantly higher than in the control group, with a similar increasing trend observed in the cerebellum, albeit without statistical significance.

### 3.6 Expression characteristics of RHBDF2 and TNFRSF10B in multiple neurodegenerative diseases

To ascertain the specificity of the heightened expression of RHBDF2 and TNFRSF10B in AD, our study extended to examining the expression profiles of these genes in various other neurodegenerative disorders, including Parkinson’s disease (PD), frontotemporal dementia (FTD), dementia with Lewy bodies (DLB), and Huntington’s disease (HD). As outlined in **Table 1**, our results demonstrate that in PD, the expression of RHBDF2 and TNFRSF10B in brain regions such as the prefrontal cortex, substantia nigra, and thalamus shows no significant deviation from normal controls, indicating that they may not serve as specific markers for PD. Similarly, we detected no noteworthy disparities in the expression of these genes in the prefrontal cortex of FTD and DLB patients in comparison to controls, further underscoring their specificity for AD. Contrastingly, in HD, a distinct expression profile was observed; the expression of RHBDF2 and TNFRSF10B was notably diminished in the prefrontal cortex of HD patients when juxtaposed with normal controls.

**Table 1:** Expression characteristics of RHBDF2 and TNFRSF10B in multiple neurodegenerative diseases.

datasets	brain region	disease	genes	logFC	adjusted p-value
GSE20168	prefrontal cortex	PD	RHBDF2	0.329	0.206
			TNFRSF10B	0.54	0.182
GSE7621	substantia nigra	PD	RHBDF2	0.493	0.491
			TNFRSF10B	0.509	0.422
GSE20291	putamen	PD	RHBDF2	-1.193	0.064
			TNFRSF10B	-1.146	0.066
GSE20292	substantia nigra	PD	RHBDF2	0.348	0.372
			TNFRSF10B	0.243	0.533
GSE195872	prefrontal cortex	FTD	RHBDF2	-0.177	0.681
			TNFRSF10B	-0.324	0.63
GSE150696	prefrontal cortex	DLB	RHBDF2	0.106	0.941
			TNFRSF10B	0.257	0.93
GSE33000	prefrontal cortex	HD	RHBDF2	-0.379	8.808E-42
			TNFRSF10B	-0.231	5.893E-33

The dataset was downloaded from the GEO database, and gene difference analysis was based on the “limma” R software package.

PD, Parkinson’s disease; FTD, Frontotemporal dementia; DLB, dementia with Lewy bodies; HD, Huntington’s disease.

### 3.7 Performance of diagnosis for AD using selected feature genes

**Figure 6:**
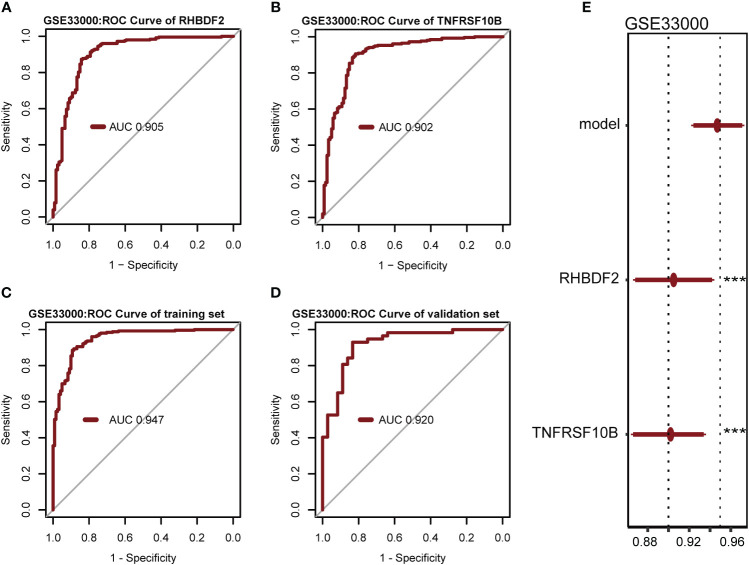
Performance of the prediction to diagnose AD in GSE33000 dataset. **(A, B)** AUROC for RHBDF2 and TNFRSF10B. **(C, D)** AUROC of multivariate model in the training set and internal validation set. **(E)** AUROC analysis between multivariate model and feature genes. Statistic tests: DeLong test. Data are presented as AUC ± 95% confidence interval [CI]. ***p < 0.001.

In evaluating the biomarker potential of each identified gene, we assessed their performance as classifiers for AD diagnosis in the training dataset. RHBDF2 and TNFRSF10B exhibited AUROC values of 0.905 (**Figure 6A**) and 0.902 (**Figure 6B**), respectively, effectively distinguishing between the control and AD groups. Subsequently, a logistic regression diagnostic model (F = 149.2, p < 0.001) was developed by merging these genes with clinical data (age and gender), with the regression coefficients detailed in **Table 2**, highlighting the substantial diagnostic contribution of RHBDF2 and TNFRSF10B in the model. Evaluation of variance inflation factors for each variable indicated values below 5, signifying absence of multicollinearity. The AUC of this comprehensive model increased to 0.947 (**Figure 6C**) in the training dataset and 0.920 (**Figure 6D**) in the internal validation dataset, demonstrating statistically significant predictive power (DeLong test p < 0.001) compared to individual genes (**Figure 6E**). Analysis of the multivariate model in the internal validation dataset revealed strong specificity identification capabilities, with recall and f1 scores exceeding 90% (**Table 3**), further affirming the efficacy of the multivariate model in accurately identifying cases.

**Table 2:** Relationships of group with feature genes and other indicators.

	coefficients	SE	p
Group ~ RHBDF2 + TNFRSF10B + Age + Gender			
RHBDF2	0.521	0.114	<0.001
TNFRSF10B	0.508	0.155	0.001
age	0.013	0.002	<0.001
gender	-0.019	0.033	0.566

Data were derived from clinical and RNA sequencing samples from the GSE33000 dataset.

SE, Standard Error.

**Table 3:** Evaluation indicators for prediction models.

accuracy	0.882
error rate	0.118
precision	0.883
specificity	0.806
recall rate	0.930
f1 score	0.906

The classification threshold is 0.5.

### 3.8 Validation of diagnosis for AD by external datasets

**Figure 7:**
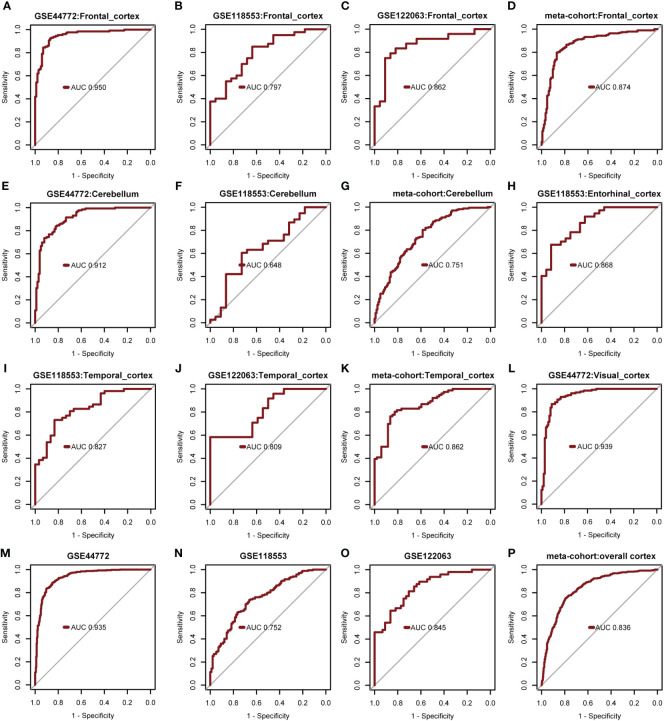
Verify performance of predictive models for diagnosing AD. **(A-D)** AUROC for multivariate modeling of frontal cortex in GSE44772, GSE118553, GSE122063, and meta-cohort (frontal cortex). **(E-G)** AUROC for multivariate modeling of cerebellum in GSE44772, GSE118553, and meta-cohort (cerebellum). **(H)** AUROC for multivariate modeling of entorhinal cortex in GSE118553. **(I-K)** AUROC for multivariate modeling of temporal cortex in GSE118553, GSE122063, and meta-cohort (temporal cortex). **(L)** AUROC for multivariate modeling of visual cortex in GSE44772. **(M-P)** AUROC for multivariate modeling in GSE44772, GSE118553, GSE122063, and meta-cohort (overall cortex).

The diagnostic model’s predictive performance underwent rigorous evaluation using three independent external validation datasets: GSE44772, GSE118553, and GSE122063 covering diverse brain regions for a comprehensive assessment of its predictive capabilities. In the frontal cortex dataset, the model exhibited medium-high accuracy with AUROC values of 0.950 (GSE44772), 0.797 (GSE118553), 0.862 (GSE122063), and 0.874 in the meta-cohort (**Figures 7A-D**). For the cerebellum dataset, the model showed moderate accuracy with AUROC values of 0.912 (GSE44772), 0.648 (GSE118553), and 0.751 in the meta-cohort (**Figures 7E-G**). In the entorhinal cortex dataset, the model performed with medium-high accuracy with an AUROC value of 0.868 (GSE118553, **Figure 7H**). Additionally, in the temporal cortex dataset, the model displayed medium-high accuracy with AUROC values of 0.827 (GSE118553), 0.809 (GSE122063), and 0.862 in the meta-cohort (**Figures 7I-K**). Notably, in the visual cortex dataset, the model showcased high accuracy, achieving an AUROC value of 0.939 (GSE44772) (**Figure 7L**). The AUROC values for GSE44772, GSE118553, GSE122063, and the merged datasets were 0.935, 0.752, 0.845, and 0.836, respectively (**Figures 7M-P**). These impressive findings affirm the model’s stable and reliable predictive ability across various brain regions, including mixed regions, for AD diagnosis.

### 3.9 Visualization of the diagnostic model for AD

**Figure 8:**
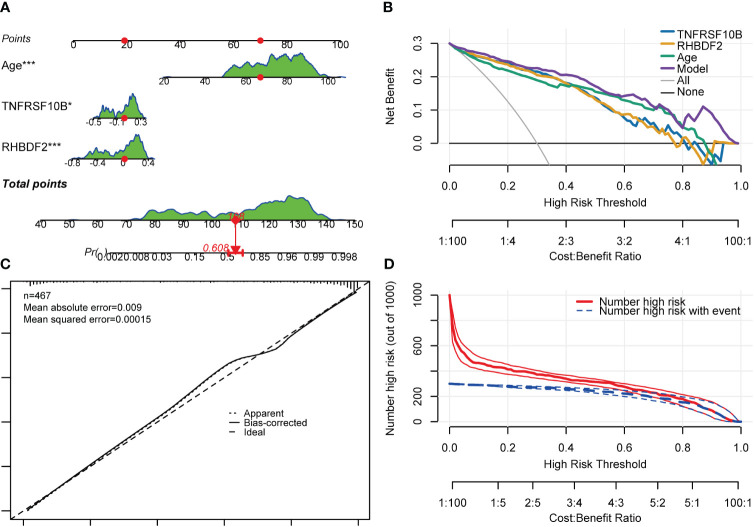
Nomogram, CCA, DCA and CIC of the multivariate diagnostic classifier. **(A)** Nomogram for evaluating the risk of AD occurrence. **(B)** DCA of the RHBDF2, TNFRSF10B, age and the multivariate diagnostic classifiers. **(C)** CCA for the relationship between the predicted probability of AD occurrence and the actual probability. **(D)** CIC of the multivariate diagnostic classifiers *p < 0.05, and ***p < 0.001.

Gender was found not to significantly contribute to the diagnosis of AD. Consequently, the model was readjusted by excluding the gender variable. Interestingly, all regression coefficients in the new model were found to be highly significant (p < 0.05). Subsequent comparison using the “anova” function indicated that the new model performed just as well as the model with all four predictor variables included (p = 0.448). A risk nomogram was then created based on this model to serve as a multivariate diagnostic classifier. Each factor (TNFRSF10B, RHBDF2, and age) was assigned specific values on the corresponding scale axis, and their scores were calculated by drawing a line. The sum of these scores gave a total score, from which a probability of AD diagnosis for each patient could be determined by drawing another line on the risk axis (**Figure 8A**). The CCA for the incidence of AD displayed a noticeable overlap between the actual and predicted incidence rates, highlighting the nomogram’s exceptional predictive value (mean absolute error = 0.009) (**Figure 8C**). Additionally, a DCA curve was employed to evaluate the performance of TNFRSF10B, RHBDF2, and age, along with the multivariate diagnostic classifier model (**Figure 8B**). The DCA illustrated that utilizing the diagnostic model for predicting AD occurrence provided more substantial benefits compared to diagnosing all or none of the patients, particularly when the threshold probability ranged from 10% to 80%. Although the net benefit within this interval was comparable, the multivariate diagnostic classifier exhibited a superior net benefit over individual diagnostic genes. Furthermore, to assess the clinical utility of the nomogram, a CIC was plotted based on the DCA results, visually showcasing the superiority of the nomogram within a broad and practical range of threshold probabilities. This positive impact on diagnosis underscored the excellent predictive value of the diagnostic model (**Figure 8D**). Similarly, the CIC for the individual diagnostic genes yielded comparable results (**Supplementary Figure S7**).

### 3.10 Small molecular drugs docking of feature genes

**Figure 9:**
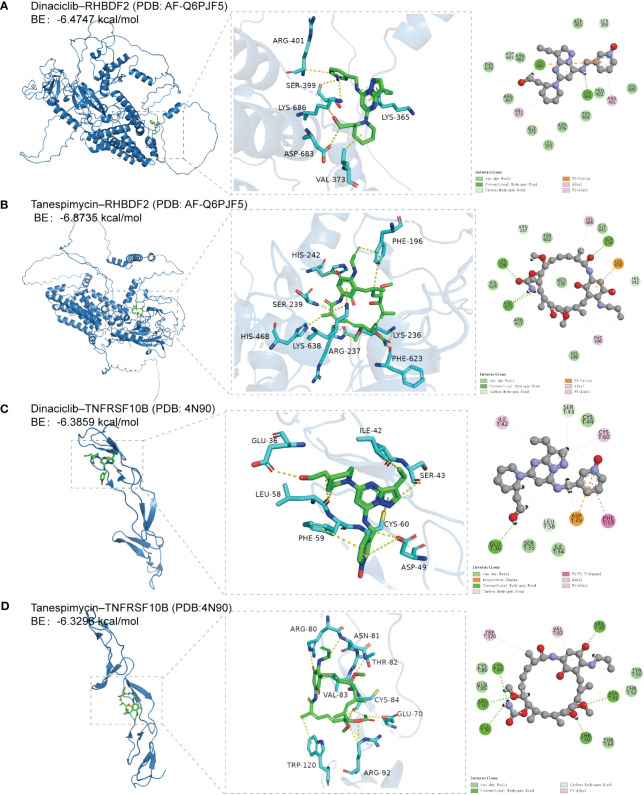
The docking results of feature genes encoded proteins with small molecular drugs. **(A)** The docking result of RHBDF2 with dinaciclib. **(B)** The docking result of RHBDF2 with tanespimycin. **(C)** The docking result of TNFRSF10B with dinaciclib. **(D)** The docking result of TNFRSF10B with tanespimycin.

RHBDF2 and TNFRSF10B have been identified as potential characteristic genes associated with AD. Therefore, small molecule drugs developed targeting these proteins may represent a promising therapeutic option for AD. 15 genes exhibiting a co-expression correlation coefficient > 0.85 with RHBDF2 and TNFRSF10B were identified from the GSE33000 dataset (**Supplementary Figure S8A**). Subsequently, using the criteria of FDR < 0.05 and connectivity score < -0.8, two small molecule drugs, dinaciclib and tanespimycin, were selected from the CMap database (**Supplementary Figure S8B**). The perturbation expression profiles of these drugs were inversely correlated with AD-related perturbation expression profiles, suggesting their potential for improving AD. To further investigate the interactions between dinaciclib, tanespimycin, and the target proteins RHBDF2 and TNFRSF10B, molecular docking studies were conducted using MOE 2019 software. The docking results indicated that dinaciclib and tanespimycin are capable of binding to the binding domains of RHBDF2 and TNFRSF10B, with molecular docking energies ranging from -6.3296 to -6.8735 kcal/mol. Based on the molecular docking results, it is evident that dinaciclib and tanespimycin exhibit strong binding energy with RHBDF2 and TNFRSF10B. Specifically, in the RHBDF2 receptor, the residues Lys686 and Lys365 interact with dinaciclib through hydrogen bonding, while the residues Asp683 and Ser399 interact with dinaciclib through carbon-hydrogen interactions. Additionally, Val373, Arg401, and Lys365 residues engage in hydrophobic interactions with dinaciclib, and the residue Lys686 forms an electrostatic interaction with the compound (**Figure 9A**, **Supplementary Figure S9A**). Within the RHBDF2 receptor, the residues Lys236, Phe623, and Ser239 form hydrogen bonds with tanespimycin, while Arg237 and His242 interact with tanespimycin through carbon-hydrogen interactions. Furthermore, the residues His468, Phe196, and Lys638 engage in hydrophobic interactions with tanespimycin, with Lys638 also binding to the compound through electrostatic interactions (**Figure 9B**; **Supplementary Figure S9B**). On the TNFRSF10B receptor, the residue Glu36 interacts with dinaciclib via hydrogen bonding, while the residues Leu58, Ser43, and Asp49 interact with dinaciclib through carbon-hydrogen interactions. Additionally, the residues Ile42, Cys60, and Phe59 participate in hydrophobic interactions with dinaciclib, and the residue Asp49 engages in electrostatic interactions with the compound (**Figure 9C**; **Supplementary Figure S9C**). The residues Cys84, Arg92, Glu70, Asn81, Thr82, and Arg80 on the TNFRSF10B receptor form hydrogen bonds with tanespimycin, while Cys84, Arg92, and Asn81 interact with tanespimycin through carbon-hydrogen bonding. Furthermore, Trp120 and Val83 residues interact with tanespimycin through hydrophobic interactions (**Figure 9D**; **Supplementary Figure S9D**). In conclusion, according to the CMap database and molecular docking results, dinaciclib and tanespimycin may represent potential options for treating AD.

## 4 Discussion

AD, a chronic neurodegenerative disease and the top cause of dementia, is influenced by a range of genetic, environmental, and lifestyle factors (35). Despite extensive research, a comprehensive understanding of AD’s pathogenesis is still lacking, and no effective treatment has been developed thus far. Given the significant variation in pathogenesis among AD patients, further investigation is necessary to explore biodiagnostic markers.

ML offers a unique approach to efficiently process multidimensional data, integrate data from various sources, and discover novel biomarkers. The application of ML in disease management and the development of therapeutic options has proven invaluable (36). In our study, we aimed to further explore the genomic characteristics of AD by utilizing the WGCNA algorithm to construct co-expression networks. Our analysis revealed the blue module as a significant gene module positively associated with AD. Functional enrichment analysis showed that the feature genes within the blue module were predominantly enriched in immune response-related functions and pathways. By employing a cut-off criteria of GS > 0.70 and MM > 0.92, we identified two feature genes, namely RHBDF2 and TNFRSF10B, that were associated with AD.

Among the immune-related genes linked to AD, RHBDF2 and TNFRSF10B show promising diagnostic potential (4). Our comparative analysis of RHBDF2 and TNFRSF10B expression in various neurodegenerative diseases has revealed that their heightened expression is distinctive to AD, contrasting conditions like PD, FTD, DLB, and HD. This observation underscores the diagnostic value of these markers in AD and their potential for clinical application. Although current research primarily relies on post-mortem brain tissue, the absence of evidence for detecting RHBDF2 and TNFRSF10B in body fluids such as peripheral blood or cerebrospinal fluid does not rule out potential future advancements. As technology progresses and research intensifies, novel methods for detecting these molecules in body fluids may emerge. More sensitive assays or advanced biomarker detection technologies, including single-molecule sequencing, could facilitate their detection in body fluid samples. Additionally, our finding that RHBDF2 and TNFRSF10B are significantly expressed in multiple brain regions - including the frontal cortex, temporal cortex, and visual cortex - provides flexibility in selecting a safe and accessible site for brain biopsy sampling. As minimally invasive techniques improve, physicians may conduct more precise and safer brain surgeries for tissue sampling, further advancing our knowledge and treatment of neurodegenerative diseases.

Considering the age-related nature of AD and the observed gender disparities in its incidence, prevalence, and biomarker profiles (37), we analyzed the expression patterns of RHBDF2 and TNFRSF10B concerning age and gender. Our findings demonstrated a consistent increase in the expression of both genes among individuals aged 65 years and older, particularly those with AD. Notably, the expression levels were significantly higher in AD patients than in controls, irrespective of age. Furthermore, we observed higher expression levels in females compared to males, a trend also evident among AD patients. These results suggest that RHBDF2 and TNFRSF10B may play a critical role in the pathogenesis of AD and are influenced by both age and gender.

To further explore the potential of RHBDF2 and TNFRSF10B as biomarkers, we integrated these genes with variables such as age and gender, employing a multivariate logistic regression model to evaluate their effectiveness in distinguishing between the control group and AD patients. The model exhibited impressive results, demonstrating outstanding performance in both the training set and internal validation set, with AUC values exceeding 0.9, indicating strong diagnostic accuracy. To further validate the diagnostic utility of these genes, we meticulously developed diagnostic nomograms and assessed the model’s predictive capacity from various perspectives using CCA, DCA, and CIC analyses. The findings from these extensive validations confirm the reliability and precision of the model, highlighting the potential of RHBDF2 and TNFRSF10B as robust biomarkers for AD.

Through comprehensive bioinformatic analysis, we observed significant upregulation of RHBDF2 in the AD patient group. RHBDF2, also known as iRhom2, belongs to the rhomboid family, which is an evolutionarily conserved family of intramembrane serine proteases (38, 39). Although structurally similar to rhomboid, RHBDF2 lacks the essential catalytic residues, earning it the classification of a pseudoprotease (38). Mammals possess two forms of iRhom, RHBDF1 (also known as iRhom1) and RHBDF2. RHBDF2 acts as a cargo receptor for a disintegrin and metalloprotease 17 (ADAM17), facilitating its trafficking from the endoplasmic reticulum to the Golgi apparatus. Within the Golgi apparatus, ADAM17 matures through furin-mediated cleavage, which removes its inhibitory structural domain. Ultimately, RHBDF2 aids in ADAM17 translocation to the plasma membrane, facilitating the shedding of TNF and epidermal growth factor receptor (EGFR) ligands (38, 40, 41). Previous studies have highlighted the diverse functions of RHBDF2 in immune-mediated diseases, specifically its regulation of pathways such as TNF, EGFR, and stimulator of interferon genes signaling (41). Our GeneMANIA analysis further supported the involvement of RHBDF2 in the EGFR and ERBB signaling pathways, where it interacts with RHBDF1, ADAM17, and EGF within the GeneMANIA network. Immune dysregulation is recognized as a significant contributor to AD pathogenesis. Epigenome-wide association studies focused on AD have identified an association between DNA methylation at RHBDF2 loci and AD risk (42–45). However, the precise mechanism by which RHBDF2 contributes to AD remains unclear. Notably, RHBDF2 operates within the same protein interaction network as PTK2B (43), a known AD risk gene that plays a crucial role in the signaling cascade governing microglia and infiltrating macrophage activation (46). Furthermore, RHBDF2 exhibits expression in a specific subset of immune cells, including microglia. This suggests that RHDBF2 might aggravate AD pathology through microglia-driven inflammatory responses (38, 47, 48). In line with this, Bennett et al. reported an increase in RHBDF2 expression in the context of AD (42). Consistently, our study’s violin diagram displayed significantly elevated gene expression levels of RHBDF2 in the AD group compared to the control group. Therefore, targeting RHBDF2 holds potential for exploring novel therapeutic interventions for AD.

We also discovered TNFSR10B as another risk gene showing upregulation in the AD group. TNFSR10B, also known as death receptor 5 (DR5) or TRAIL-R2, acts as a receptor for TNF-related apoptosis-inducing ligand (TRAIL), which is also referred to as Apo-2 ligand (Apo2L), and TNFSF10 (49, 50). The initial report by Rauch et al. (51). in 1997 introduced TRAIL as a potent pro-apoptotic cytokine that induces apoptosis during peripheral and central inflammation through complex interactions between ligands and receptors (49, 50, 52). The TNF superfamily includes five distinct TRAIL receptors: DR4 (TRAIL-R1, TNFRSF10A), DR5 (TNFRSF10B), DcR1 (TRAIL-R3, TNFRSF10C), DcR2 (TRAIL-R4, TNFRSF10D), and osteoprotegerin (49, 52). Notably, DR4 and DR5 activate the extrinsic apoptosis pathway through their intracellular death domains, while DcR1 and DcR2 function as decoy receptors (49, 50, 52). Consistent with these findings, our GeneMANIA analysis indicated that TNFRSF10B is primarily involved in the extrinsic apoptotic signaling pathway, interacting with TNFSF10, FAS, FADD, and CASP8 to form the death-inducing signaling complex. Numerous *in vivo* and *in vitro* studies have reported the involvement of TNFSF10/TNFRSF10B in amyloid β peptide (Aβ)-induced neurotoxicity (50, 53–55). Immunoneutralization of TNFSF10 or blockade of TNFRSF10B has been shown to prevent Aβ-mediated neurotoxicity and suppress the immune/inflammatory response, as confirmed in both *in vivo* and *in vitro* experiments (50, 53–55). Thus, the interaction between TNFSF10 and TNFRSF10B holds promise as a potential therapeutic target for AD.

While previous research has suggested the pathological roles of RHBDF2 and TNFRSF10B in the progression of AD, there has been a lack of in-depth exploration into their specific connections with immune cells or immune responses. Particularly regarding the role of RHBDF2 in AD, current studies are relatively limited. Existing clues suggest that RHBDF2 may exacerbate the pathology of AD through inflammation driven by microglia, as indicated by its extension through the same protein interaction network as PTK2B. Our research further confirms the detrimental impacts of elevated levels of RHBDF2 and TNFRSF10B in the development of AD, linking them to immune cells, especially microglia, providing a new research direction for these factors in exacerbating AD pathology through mediating immune dysregulation. Notably, our study also reveals a close association of RHBDF2 and TNFRSF10B with immune cell infiltration in AD progression. Within AD patients, levels of monocytes, neutrophils, M1 macrophages are significantly increased, while levels of plasma cells and CD8+ T cells are decreased. Correlation analyses indicate that the expression levels of RHBDF2 and TNFRSF10B are positively correlated with monocytes, neutrophils, and M1 macrophages, and negatively correlated with CD8+ T cells and plasma cells. Overall, these findings suggest that the dysregulation or dysfunction of RHBDF2 and TNFRSF10B may be pathogenic factors in AD, likely exacerbating the pathological process of AD through immune and inflammatory reactions mediated by microglia or peripheral infiltrating immune cells.

Our study paves the way for unravelling the molecular mechanisms of immune dysregulation in AD. Ultimately, our aim is to develop personalized neuroimmune therapeutic strategies that actively impact the treatment outcomes of AD by targeting the interactions between RHBDF2, TNFRSF10B, and immune cells. To align with the development of novel therapeutic approaches, we screened 17 specific genes, including RHBDF2 and TNFRSF10B, in the CMap database to identify potential AD therapeutic drugs. Notably, we identified dinaciclib, a cyclin-dependent kinase (CDK) inhibitor, and tanespimycin, a heat shock protein (HSP) inhibitor, as potential candidate drugs for treating AD targeting RHBDF2 and TNFRSF10B. In addition, molecular docking results showed that dinaciclib and tanespimycin had better binding activity with RHBDF2 and TNFRSF10B.

CDKs are key regulators of the eukaryotic cell cycle and are involved in essential biological processes such as transcription, metabolism, communication, and apoptosis (56). Recent studies have reported the association between cell cycle dysregulation and key pathological features of AD, including the accumulation of amyloid-beta (Aβ) deposits and hyperphosphorylated tau (57). It has been proposed that cell cycle re-entry contributes to neuronal death in AD (58), thereby suggesting CDKs as potential therapeutic targets (57). The potential use of dinaciclib, a multi-CDK inhibitor targeting CDK1, 2, 5, and 9, in the treatment of AD is yet to be evaluated.

HSPs, a family of highly conserved molecular chaperones, play a crucial role in maintaining intracellular protein homeostasis. They accomplish this by ensuring proper folding, assisting in the refolding of denatured proteins, and promoting the degradation of damaged proteins (59). Among the HSPs, HSP90 stands out as the most abundant molecular chaperone in cells. It is involved in a wide range of cellular processes, including diverse signaling and regulatory pathways (60). As a result, HSP90 has become an attractive target for drug development. One of the key functions of HSP90 is its strict regulation of heat shock factor 1 (HSF-1) activation. Normally, HSF-1 binds to HSP90 under normal conditions. However, when cells are subjected to stress, HSF-1 dissociates from HSP90. Once released, phosphorylated HSF-1 translocates to the nucleus where it regulates the transcriptional activation of various heat shock proteins, including HSP27, HSP40, HSP70, and HSP90 (61). Geldanamycin, an inhibitor of HSP90, facilitates the dissociation of the HSF-1-HSP90 complex, leading to the activation of the heat shock response. This activation is achieved through the upregulation of HSP40, HSP70, and HSP90 (62). Tanespimycin, also known as 17-(allylamino)-17-demethoxygeldanamycin, is a semi-synthetic derivative of geldanamycin that has attracted considerable attention for the treatment of various diseases, including AD. Tanespimycin possesses superior pharmacokinetics and is associated with lower toxicity. Animal studies investigating the effectiveness of tanespimycin in AD models have yielded positive results, further supporting its potential as a therapeutic agent (63–65).

In summary, the objective of this study was to delve into the molecular mechanisms underlying the pathogenesis of AD through bioinformatics and ML analysis. Our findings indicate that RHBDF2 and TNFRSF10B could play a crucial role in AD pathogenesis by disrupting the immune homeostasis of the intracerebral microenvironment via central immune cells or immune cells infiltrating from peripheral sources. These genes offer promising avenues for therapeutic research in AD and could potentially serve as diagnostic biomarkers. However, it is imperative to acknowledge the limitations of our study. Firstly, detection of RHBDF2 and TNFRSF10B expression levels in peripheral blood samples, as well as confirmation in cellular or animal models, has not yet been achieved. Furthermore, the therapeutic effects of small molecule drugs on AD have not been validated through cellular experiments. In future research, we aim to thoroughly investigate the mRNA and protein levels of these feature genes using techniques such as qPCR and western blotting. By employing gene interference methods like siRNA, we intend to elucidate the precise mechanisms of these genes in AD pathogenesis. Additionally, visualizing the binding of small molecule drugs to the target genes and their therapeutic impact on AD will necessitate conducting relevant cellular experiments to enhance the depth and comprehensiveness of our research findings. Secondly, there exist challenges in comprehending immune cell infiltration in AD brain tissue and its association with these feature genes. Future studies could validate our experimental results by performing scRNAseq/snRNAseq on physical samples. Lastly, the absence of mild cognitive impairment samples hinders the determination of whether these genes can be utilized as early diagnostic biomarkers for AD. Consequently, further basic and clinical research is imperative to validate our current understanding of the relationship between these genes and the immune system in the context of AD pathophysiology.

## 5 Conclusions

Our analysis comprehensively elucidates the intricate connection between immune dysregulation and the pathogenesis of AD. Notably, our study identifies two pivotal diagnostic effector genes, RHBDF2 and TNFRSF10B, which are intricately linked to diverse immune responses and distinct immune cell populations. Furthermore, we have successfully constructed a diagnostic model for enhanced AD diagnosis based on RHBDF2 and TNFRSF10B. Ultimately, the identification of these diagnostic genes is anticipated to propel the advancement of innovative therapeutic strategies and pave the way for targeted AD therapy, forging new frontiers in the field.

## Data availability statement

The datasets presented in this study can be found in online repositories. The names of the repository/repositories and accession number(s) can be found in the article/**Supplementary Material**.

## Author contributions

GZ: Writing – original draft, Data curation. SS: Formal analysis, Writing – original draft, Data curation. YW: Methodology, Writing – review & editing. YZ: Writing – review & editing. LS: Writing – review & editing, Supervision.
