# Supplementary material for: Unveiling Immune-related feature genes for Alzheimer’s disease based on machine learning
Source: Front Immunol. 2024 Jun 10;15:1333666. doi: 10.3389/fimmu.2024.1333666 (PMC11194375; doi:10.3389/fimmu.2024.1333666)
Supplement: Supplementary file 1 [file DataSheet_1.docx]

Supplementary Material

# Supplementary Figures and Tables

## Supplementary Figures


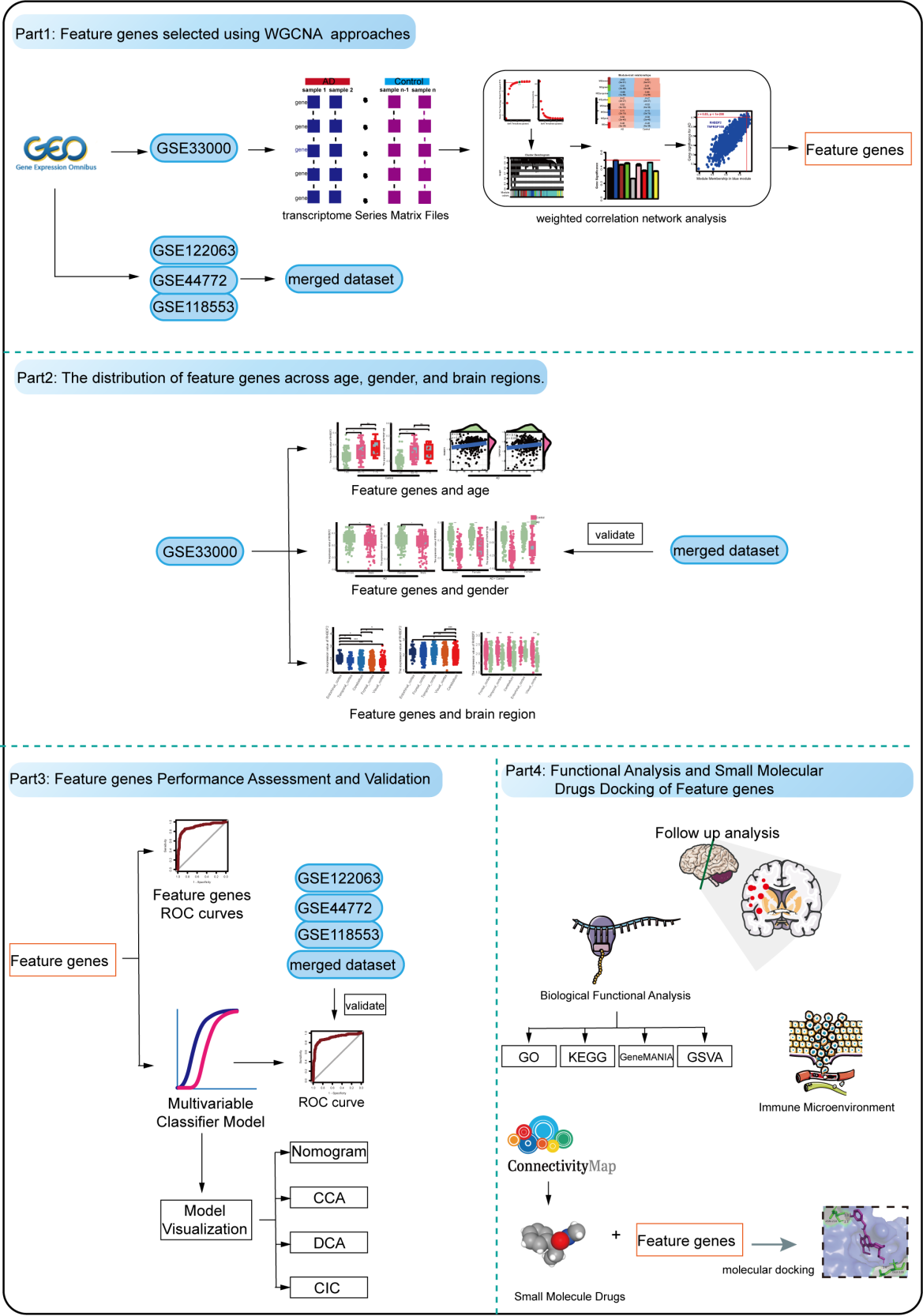


**Figure S1**. The overall design of this study. The WGCNA algorithm was used to obtain the feature genes, and the distribution of expression of the feature genes in the clinical data such as age, gender and brain region was then investigated. A multivariate diagnostic model was constructed and visualized using the feature genes in combination with the clinical data. Biofunction analysis and prediction of potential small molecule drugs were then performed on the feature genes.


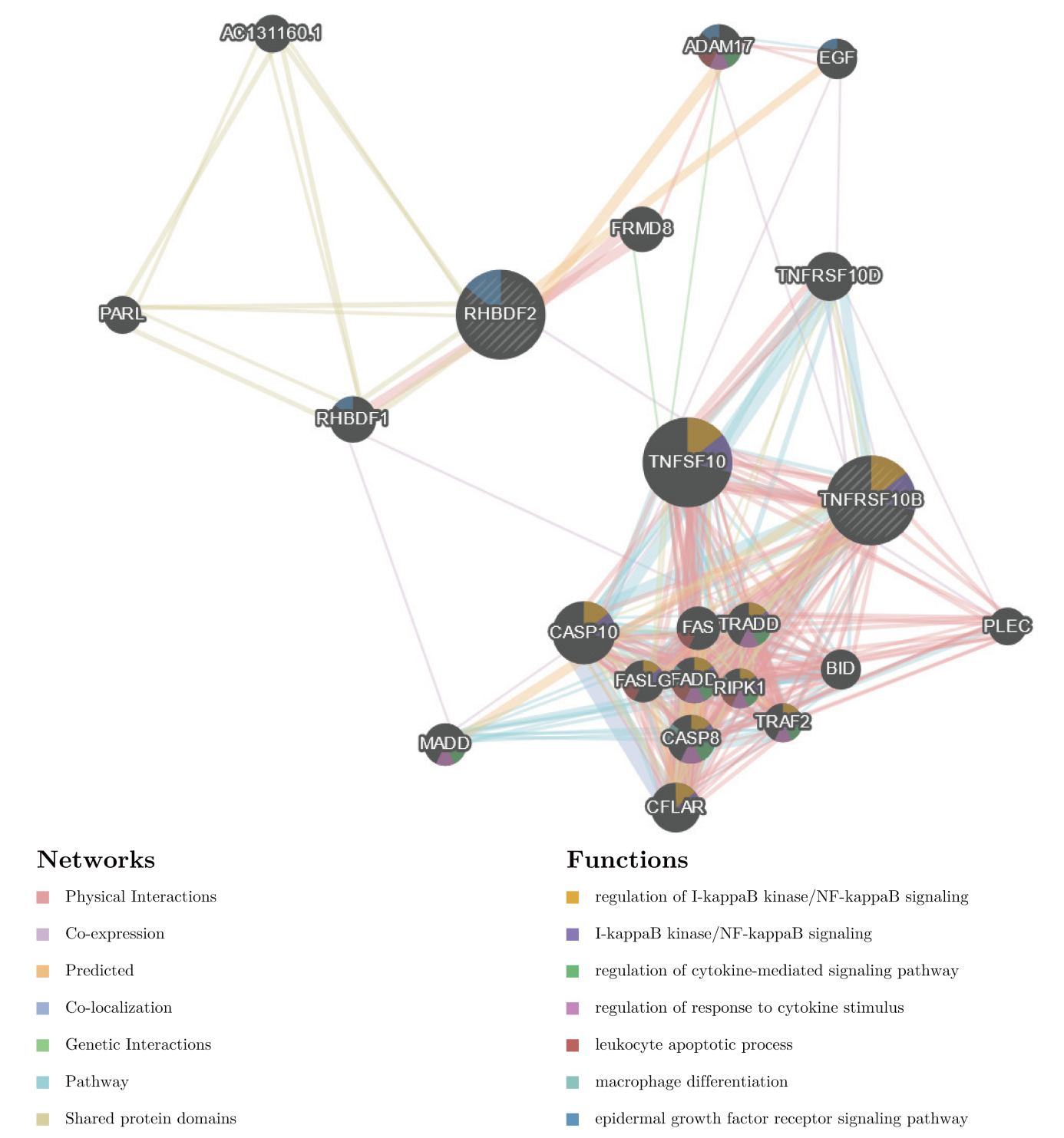


**Figure S2**. Construction of RHBDF2 and TNFRSF10B Functional Networks Using the GeneMANIA Database.


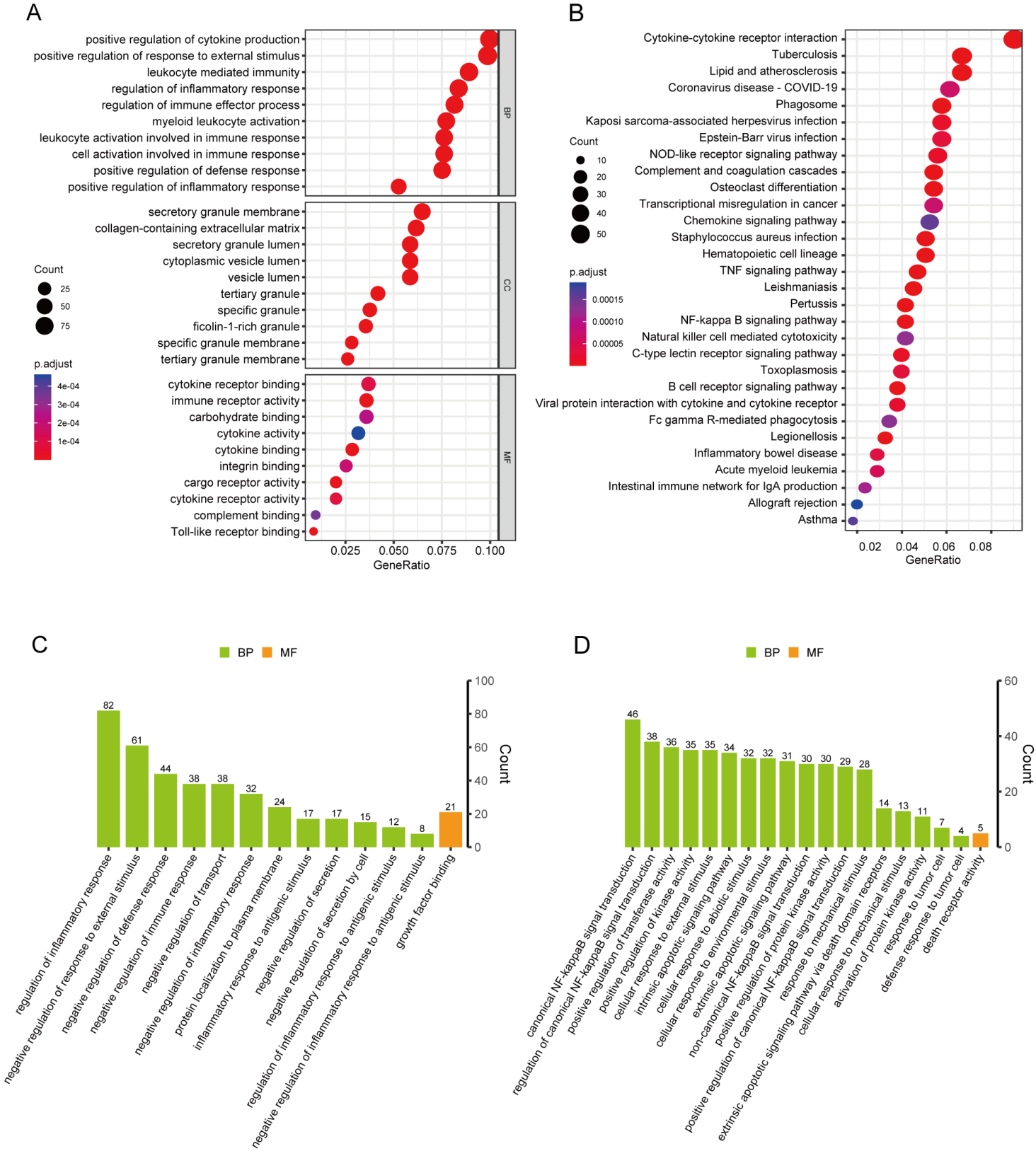


**Figure S3**. GO ontology analysis and KEGG pathway analysis. (**A**) Bubble plots of GO ontology. (**B**) Bubble plots of KEGG pathway. Bar graphs of functional enrichment analyses for potential involvement of RHBDF2 (**C**) and TNFRSF10B (**D**) in AD biological functions.


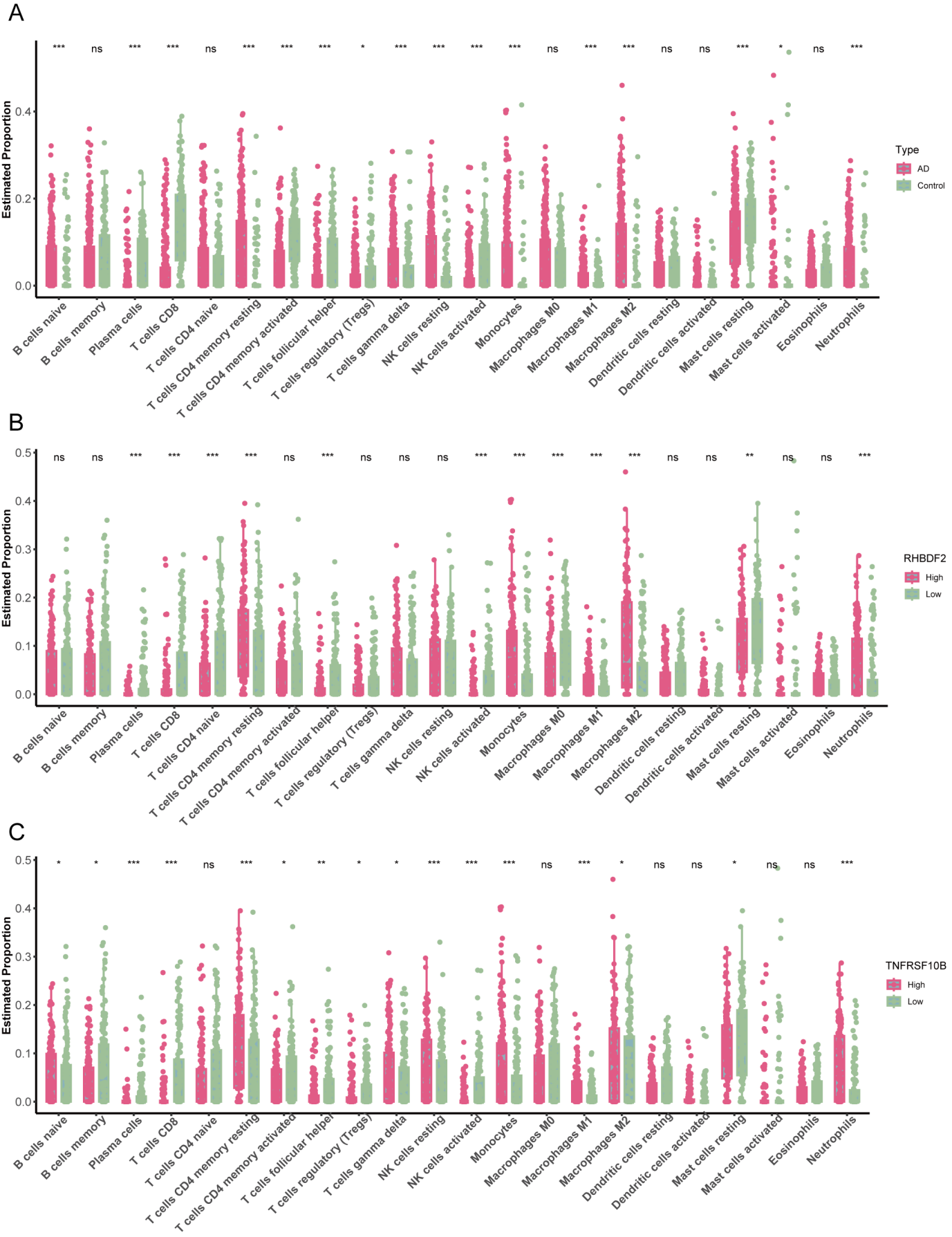


**Figure S4**. Immune cell infiltration using the CIBERSORT algorithm. (**A**) Differential analysis of immune cell proportions between AD and control groups. (**B**) Differential analysis of immune cell proportions between high and low RHBDF2 expression. (**C**) Differential analysis of immune cell proportions between high and low TNFRSF10B expression. *p < 0.05, **p < 0.01, ***p < 0.001, and ns indicates no statistical significance.


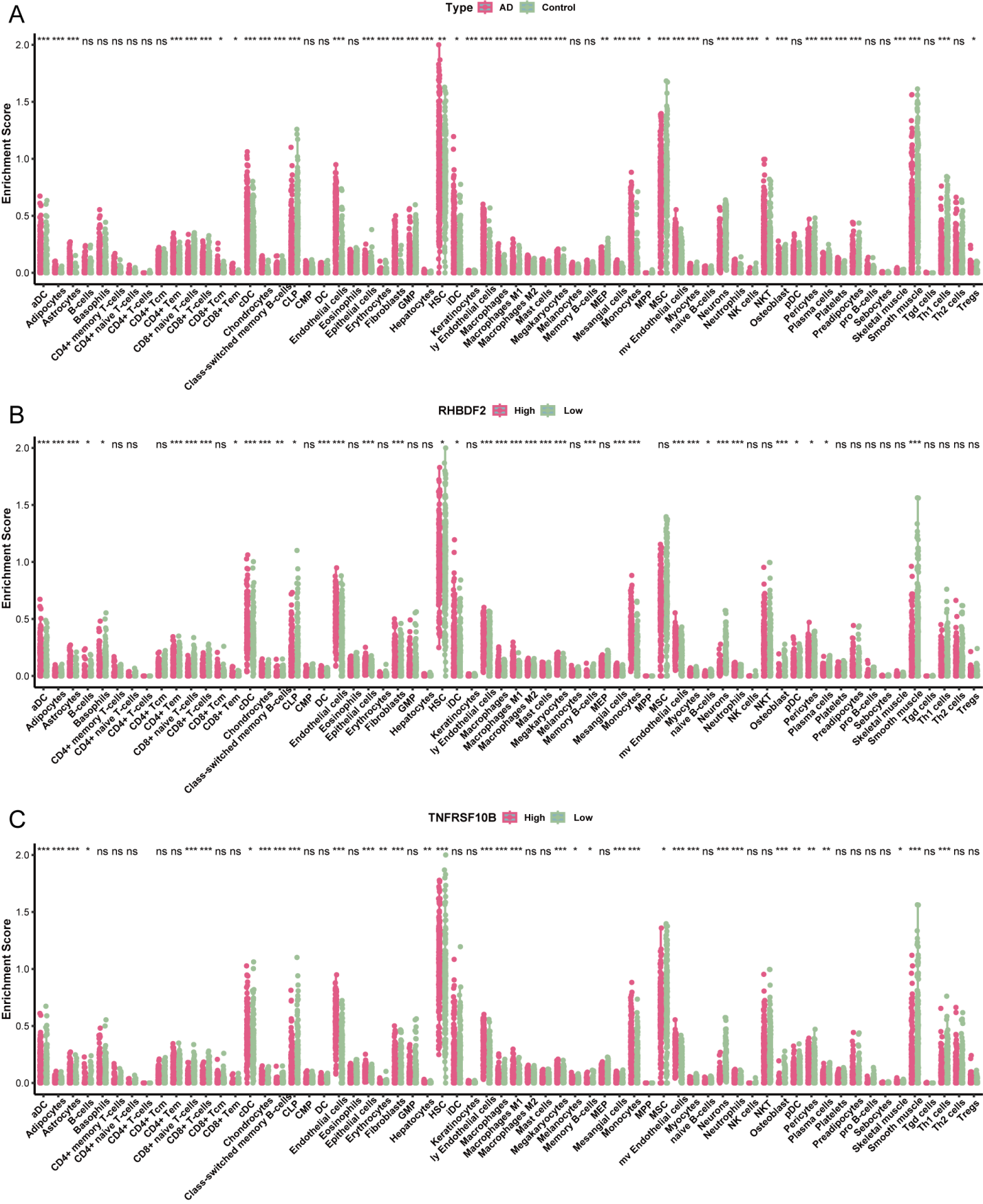


**Figure S5**. Immune cell infiltration using the xCell algorithm. (**A**) Differential analysis of immune cell enrichment score between AD and control groups. (**B**) Differential analysis of immune cell enrichment score between high and low RHBDF2 expression. (**C**) Differential analysis of immune cell enrichment score between high and low TNFRSF10B expression. *p < 0.05, **p < 0.01, ***p < 0.001, and ns indicates no statistical significance.


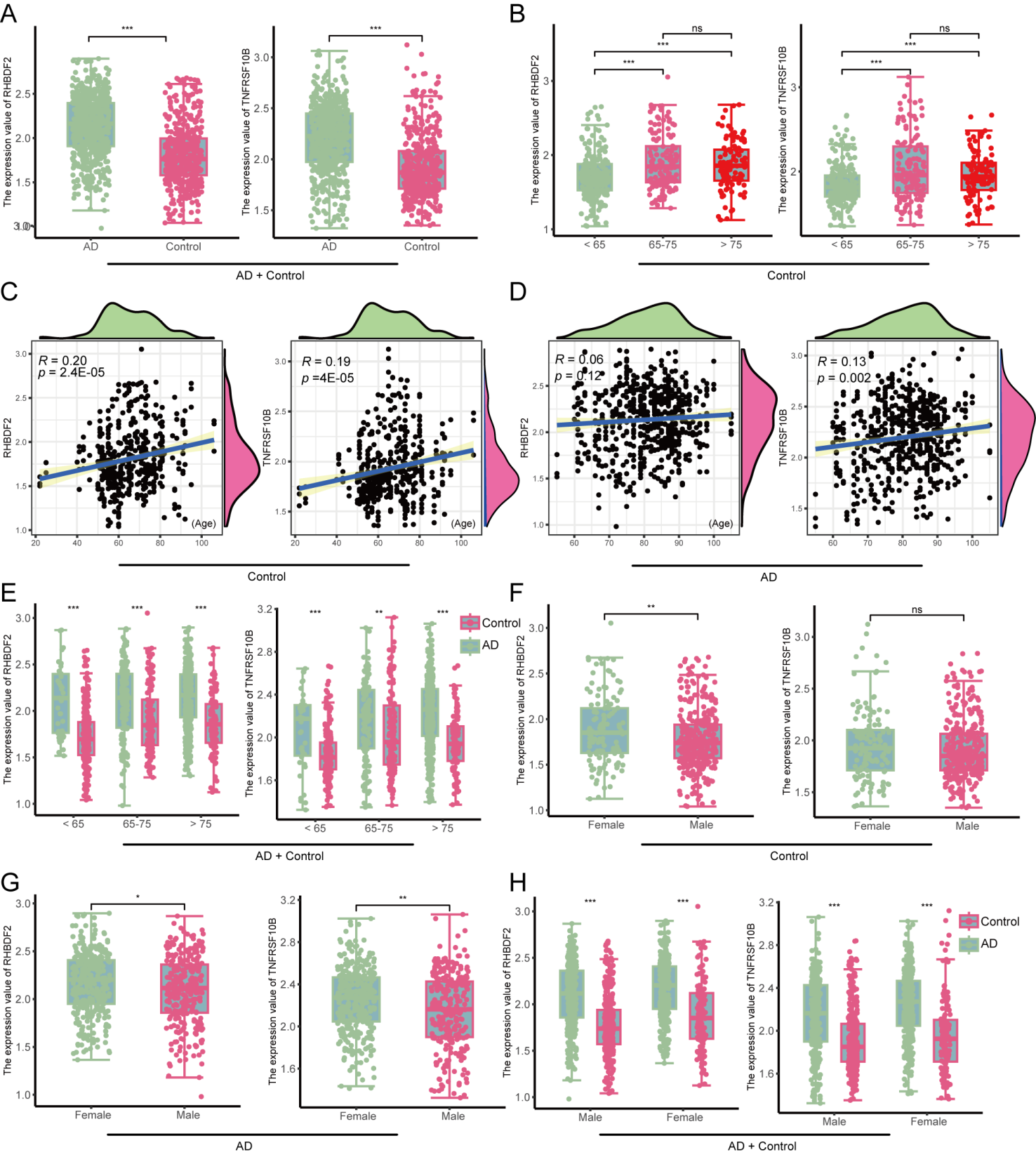


**Figure S6**. Expression of RHBDF2 and TNFRSF10B in the merged dataset was associated with age and gender. **(A)** Differential analysis of RHBDF2 and TNFRSF10B expression between the AD group and the control group. **(B)** Differential analysis of RHBDF2 and TNFRSF10B expression across different age groups in the control group. Correlation analysis of RHBDF2 and TNFRSF10B expression with age in the control group **(C)** and the AD group **(D)**. **(E)** Differential analysis of RHBDF2 and TNFRSF10B expression between the AD group and the control group across different age groups. Differential analysis of RHBDF2 and TNFRSF10B expression between genders in the control group **(F)** and the AD group **(G)**. **(H)** Differential analysis of RHBDF2 and TNFRSF10B expression between AD group and control group by gender. *p < 0.05, **p < 0.01, ***p < 0.001, and ns indicates no statistical significance.


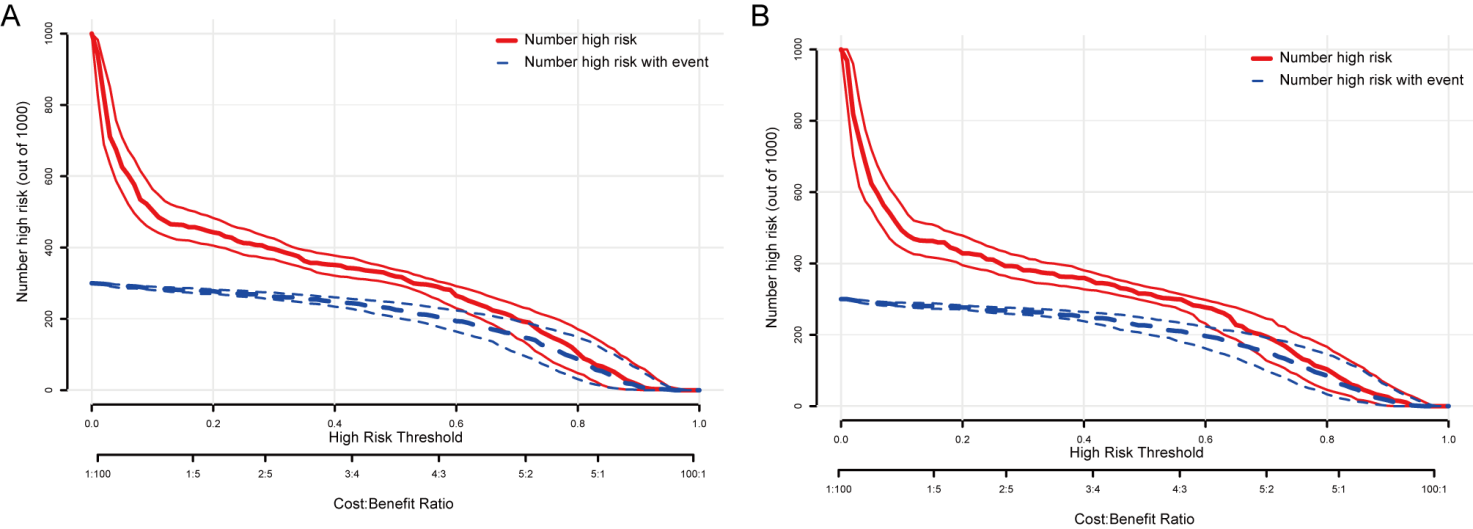


**Figure S7**. CIC used to assess the performance of individual diagnostic genes. (**A**) The CIC of RHBDF2. (**B**) The CIC of TNFRSF10B.


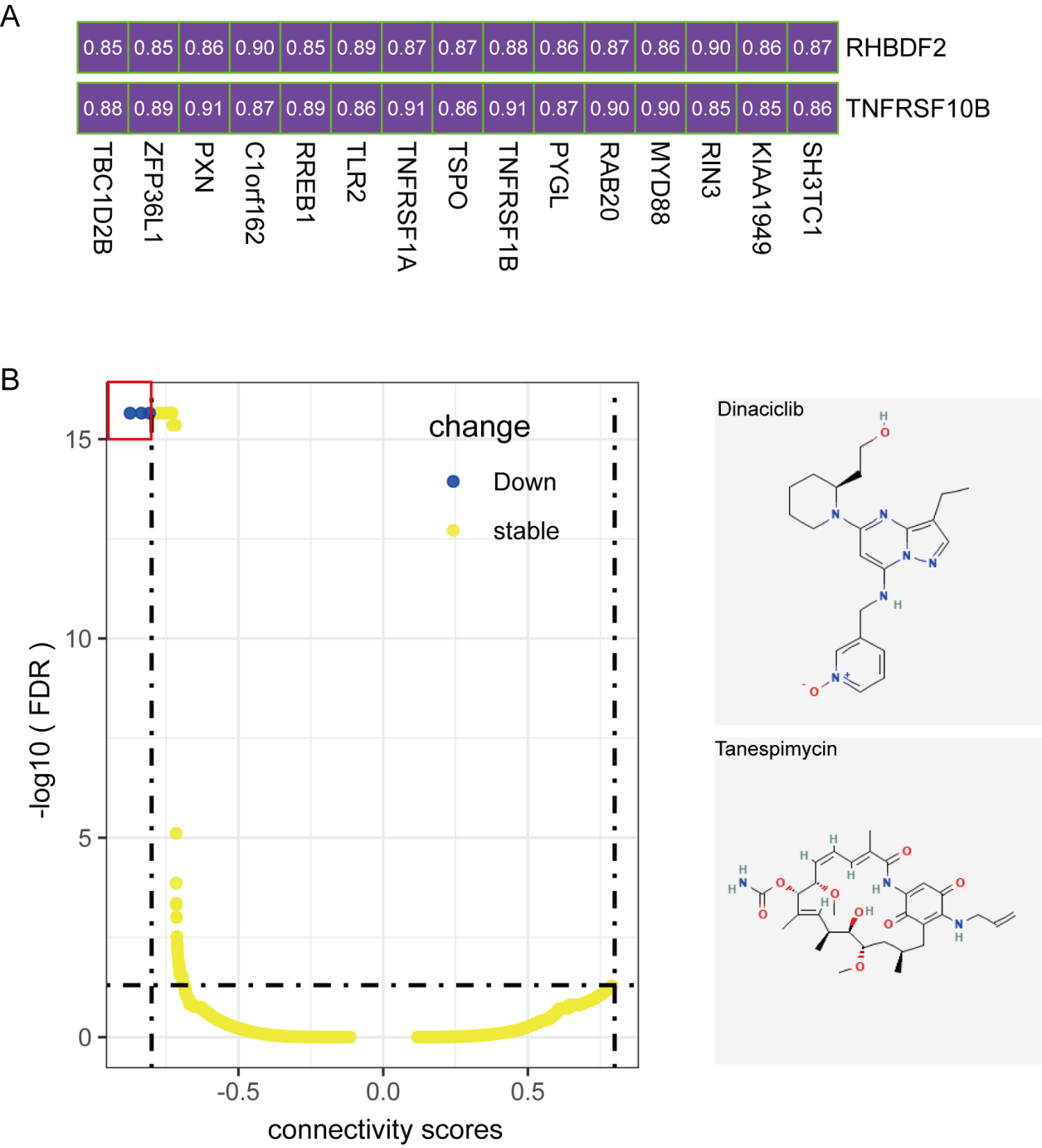


**Figure S8**. Screening of potential therapeutic drugs. (**A**) Heatmap of genes with a correlation coefficient > 0.85 with hub genes in AD patients. (**B**) Volcano plot for small molecule drugs **(left**). Dinaciclib and tanespimycin may have therapeutic potential for AD. The 2D Structure of Dinaciclib and Tanespimicin (**right**).


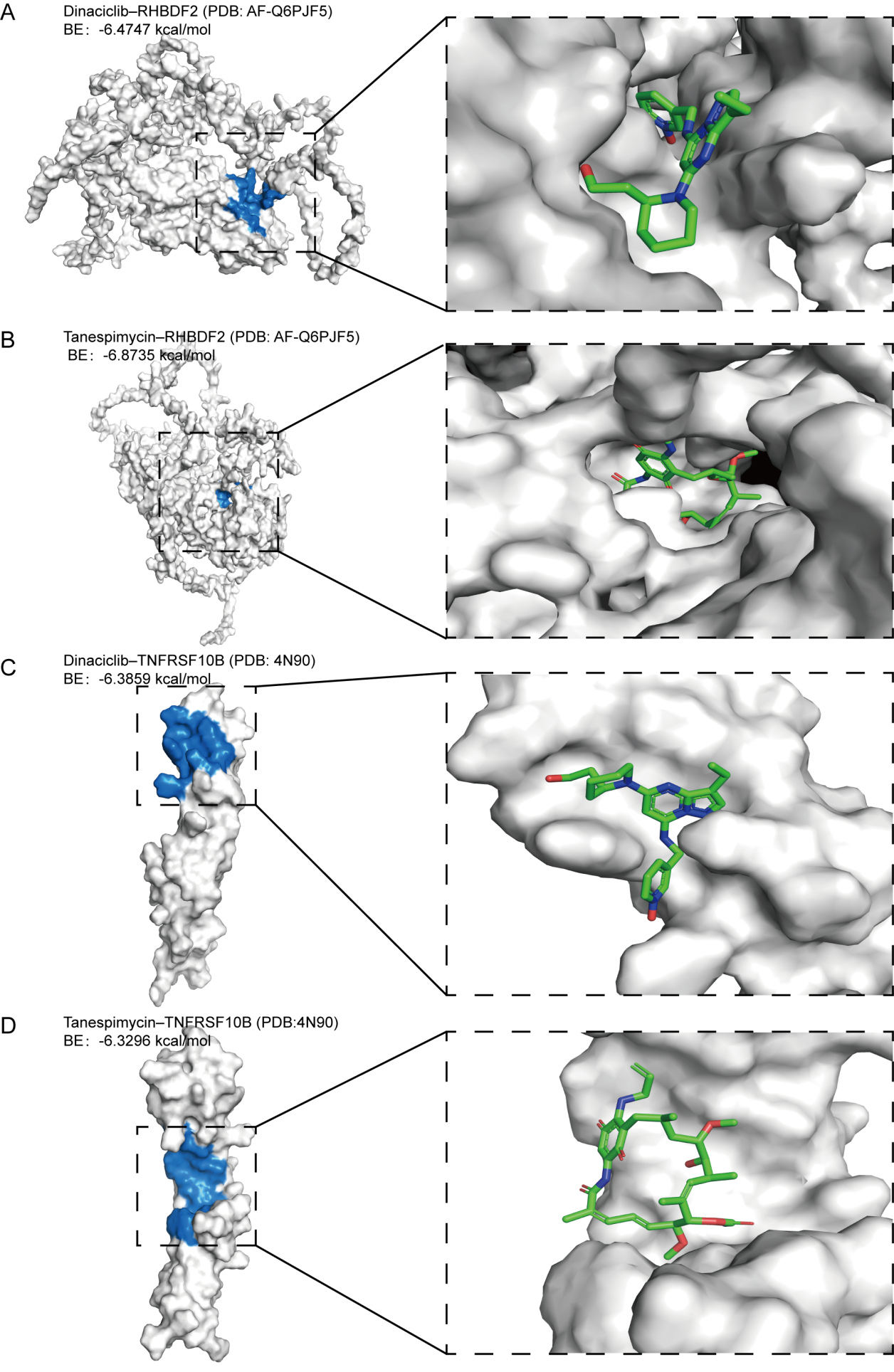


**Figure S9**. The docking results of feature genes encoded proteins with small molecular drugs. (**A**) The docking result of RHBDF2 with dinaciclib. (**B**) The docking result of RHBDF2 with tanespimycin. (**C**) The docking result of TNFRSF10B with dinaciclib. (**D**) The docking result of TNFRSF10B with tanespimycin.

## Supplementary Tables

**Table S1.** Clinical data of the dataset

|  | | | AD | Control |
| --- | --- | --- | --- | --- |
| GSE44772 | Platform | GPL4372 [Rosetta/Merck Human 44k 1.1 microarray] | | |
|  |  | Samples | 387 | 303 |
|  |  | Age | 80.14±9.24 | 62.12±10.83 |
|  | Gender | Male | 186 | 246 |
|  |  | Female | 201 | 57 |
|  | Region | Cerebellum | 129 | 101 |
|  |  | Frontal cortex | 129 | 101 |
|  |  | Visual cortex | 129 | 101 |
| GSE118553 | Platform | GPL10558 [Illumina HumanHT-12 V4.0 expression beadchip] | | |
|  |  | Samples | 167 | 98 |
|  |  | Age | 82.92±10.20 | 70.44±15.79 |
|  | Gender | Male | 69 | 55 |
|  |  | Female | 98 | 43 |
|  | Region | Cerebellum | 38 | 22 |
|  |  | Frontal cortex | 40 | 22 |
|  |  | Entorhinal cortex | 37 | 24 |
|  |  | Temporal cortex | 52 | 30 |

**Table S1.** Clinical data of the dataset (Continued)

|  | |  | |  | | AD | | Control |
| --- | --- | --- | --- | --- | --- | --- | --- | --- |
| GSE122063 | Platform | | GPL16699 [Agilent-039494 SurePrint G3 Human GE v2 8x60K Microarray] | | | | | |
|  |  | | Age | | 80.92±7.38 | | 78.82±8.84 | |
|  | Gender | | Male | | 3 | | 5 | |
|  |  |  | Female | | 9 | | 6 | |
|  | Region | | Temporal cortex | | 12 | | 11 | |
|  |  |  | Frontal cortex | | 12 | | 11 | |
|  |  | | Total samples | | 24 | | 22 | |
| GSE33000 | Platform | | GPL4372 [Rosetta/Merck Human 44k 1.1 microarray] | | | | | |
|  |  | | Samples | | 310 | | 157 | |
|  |  | | Age | | 80.60±8.99 | | 63.52±9.91 | |
|  | Gender | | Male | | 135 | | 123 | |
|  |  |  | Female | | 175 | | 34 | |
|  | Region | | Frontal cortex | | 310 | | 157 | |

**Table S2.** Interactions network of hub genes using GeneMANIA

| **Genes** | **Functions** | **Related-genes in network** |
| --- | --- | --- |
| TNFRSF10B | cellular response to abiotic stimulus | ZFP36L1, CASP8, FADD, FAS |
|  | cellular response to environmental stimulus, | ZFP36L1, CASP8, FADD, FAS |
|  | cellular response to external stimulus | CASP8, FADD, FAS |
|  | extrinsic apoptotic signaling pathway | TNFSF10, CFLAR, CASP8, FADD, TRADD, FAS, MADD, FASLG, BID |
|  | extrinsic apoptotic signaling pathway via death domain receptors | TNFSF10, CFLAR, CASP8, FADD, TRADD, FAS, MADD, FASLG |
|  | I-κB kinase/NF-κB signaling | TNFSF10, CASP10, CFLAR, CASP8, FADD, TRADD, FASLG |
|  | negative regulation of apoptotic signaling pathway | TNFSF10, CFLAR, CASP8, FADD, TRADD, FAS, FASLG |
|  | negative regulation of extrinsic apoptotic signaling pathway | TNFSF10, CFLAR, CASP8, FADD, TRADD, FAS, FASLG |
|  | positive regulation of cysteine-type endopeptidase activity | TNFSF10, CASP10, CFLAR, CASP8, FADD, TRADD, FAS, FASLG, BID |
|  | positive regulation of cysteine-type endopeptidase activity involved in apoptotic process | TNFSF10, CASP10, CFLAR, CASP8, FADD, TRADD, FAS, FASLG, BID |
|  | positive regulation of endopeptidase activity | TNFSF10, CASP10, CFLAR, CASP8, FADD, TRADD, FAS, FASLG, BID |
|  | positive regulation of peptidase activity | TNFSF10, CASP10, CFLAR, CASP8, FADD, TRADD, FAS, FASLG, BID |
|  | positive regulation of proteolysis | TNFSF10, CASP10, CFLAR, CASP8, FADD, TRADD, FAS, FASLG, BID |
|  | regulation of apoptotic signaling pathway | TNFSF10, CFLAR, CASP8, FADD, TRADD, FAS, MADD, FASLG, BID |
|  | regulation of cysteine-type endopeptidase activity | TNFSF10, CASP10, CFLAR, CASP8, FADD, TRADD, FAS, FASLG, BID |

**Table S2.** Interactions network of hub genes using GeneMANIA (Continued)

| **Genes** | **Functions** | **Related-genes in network** |
| --- | --- | --- |
|  | regulation of cysteine-type endopeptidase activity involved in apoptotic process | TNFSF10, CASP10, CFLAR, CASP8, FADD, TRADD, FAS, FASLG, BID |
|  | regulation of endopeptidase activity | TNFSF10, CASP10, CFLAR, CASP8, FADD, TRADD, FAS, FASLG, BID |
|  | regulation of extrinsic apoptotic signaling pathway | TNFSF10, CFLAR, CASP8, FADD, TRADD, FAS, MADD, FASLG, BID |
|  | regulation of extrinsic apoptotic signaling pathway via death domain receptors | TNFSF10, CFLAR, CASP8, FADD, TRADD, FAS, FASLG |
|  | regulation of I-κB kinase/NF-κB signaling | TNFSF10, CASP10, CFLAR, CASP8, FADD, TRADD, FASLG |
|  | regulation of peptidase activity | TNFSF10, CASP10, CFLAR, CASP8, FADD, TRADD, FAS, FASLG, BID |
|  | response to mechanical stimulus | CASP8, FADD, FAS |
| RHBDF2 | epidermal growth factor receptor signaling pathway | RHBDF1, ADAM17, EGF |
|  | ERBB signaling pathway | RHBDF1, ADAM17, EGF |
|  | regulation of ERBB signaling pathway | RHBDF1, ADAM17, EGF |
